# Functional Study of Cytochrome P450 Enzymes from the Brown Planthopper (*Nilaparvata lugens* Stål) to Analyze Its Adaptation to BPH-Resistant Rice

**DOI:** 10.3389/fphys.2017.00972

**Published:** 2017-11-30

**Authors:** Lei Peng, Yan Zhao, Huiying Wang, Chengpan Song, Xinxin Shangguan, Yinhua Ma, Lili Zhu, Guangcun He

**Affiliations:** ^1^State Key Laboratory of Hybrid Rice, College of Life Sciences, Wuhan University, Wuhan, China; ^2^College of Life Sciences, Guizhou Normal University, Guiyang, China

**Keywords:** rice, brown planthopper, cytochrome P450, *NlCPR*, *CYP4C61*

## Abstract

Plant-insect interactions constitute a complex of system, whereby plants synthesize toxic compounds as the main defense strategy to combat herbivore assault, and insects deploy detoxification systems to cope with toxic plant compounds. Cytochrom P450s are among the main detoxification enzymes employed by insects to combat the chemical defenses of host plants. In this study, we used *Nilaparvata lugens* (BPH) to constitute an ideal system for studying plant-insect interactions. By feeding BPHs with artificial diets containing ethanol extracts, we show that biotype Y BPHs have a greater ability to metabolize exogenous substrates than biotype 1 BPHs. *NlCPR* knockdown inhibited the ability of BPHs to feed on YHY15. qRT-PCR was used to screen genes in the P450 family, and upregulation of *CYP4C61, CYP6AX1*, and *CYP6AY1* induced by YHY15 was investigated. When the three P450 genes were knocked down, only *CYP4C61* dsRNA treatment was inhibited the ability of BPHs to feed on YHY15. These results indicate that BPH P450 enzymes are a key factor in the physiological functions of BPH when feeding on BPH-resistant rice.

## Introduction

The evolutionary arms race between plants and herbivores force these two organisms to constantly develop strategies to defend against the other for survival. To reduce insect attack, plants produce various varieties of secondary metabolites, both constitutive and induced to defend the herbivore attack (Wittstock and Gershenzon, 2002; Senthil-Nathan, 2013). Insects face an array of plant defensive toxic compounds during their feeding, and they have therefore developed various methods to overcome plant defenses, or even use plant defenses for their own benefit, to survive in the antagonistic or toxic environment of their host plants (Senthil-Nathan et al., 2009a).

Insect detoxification systems evolve during insect-plant interactions via ubiquitous enzymes, such as cytochrome P450 monooxygenases (P450s or CYPs for encoding genes), to adapt to plant secondary compounds (Heidel-Fischer and Vogel, 2015). P450 proteins function in concert with their electron transfer partners, including cytochrome P450 reductase (CPR) and cytochrome b5 (cyt b5) (Paine et al., 2005). Many insect P450s metabolize a wide range of plant allelochemicals. For example, honey bee CYP9Q3 has confirmed activity against quercetin, a flavonoid ubiquitous in honey (Mao et al., 2011), and P450 (CYP6CY3) allows a tobacco-adapted peach-potato aphid race to efficiently detoxify nicotine (Bass et al., 2013). In addition, *CYP9T2* from bark beetles encodes a myrcene hydroxylase that hydroxylates myrcene to ipsdienol (Sandstrom et al., 2006). CYP6B33 from *Papilio polyxenes* metabolizes six furanocoumarins (Mao et al., 2007), CYP6B1 from *Helicoverpa zea* metabolizes two allelochemicals (xanthotoxin and flavone), and CYP6B8 metabolizes six biosynthetically diverse plant allelochemicals (xanthotoxin, quercetin, flavone, chlorogenic acid, indole-3-carbinol, and rutin) (Li et al., 2004).

In this study, we employed a specialist pest, the brown planthopper (BPH) [*Nilaparvata lugens* Stål (Hemiptera: Delphacidae)], and its host plant rice (*Oryza sativa* L.) as a model study system. BPH is one of the most serious insect pests of rice in Asia (Kiritani, 1979; Sogawa, 1982). The BPH is a sucking insect that remove plant sap from phloem cells. The removal of plant sap and the blockage of phloem vessels by the feeding tube sheaths cause tillers to wilt, dry and turn brown, a condition called hopper burn (Sogawa, 1982; Senthil-Nathan et al., 2009b). Since the first BPH-resistant rice variety was discovered in 1969, more than 30 major BPH-resistant loci have been reported, and 13 genes have been successfully cloned (Jing et al., 2017). We used the resistant rice variety YHY15, which contains the BPH-resistance gene *BPH15* (Yang et al., 2004); although *BPH15* candidate genes do not belong to the NB-LRR family of proteins, they may be involved in a unique resistance mechanism (Lv et al., 2014). Resistance genes inhibit BPH feeding behavior and affect BPH physiology by lowering survival rates, prolonging nymphal periods, lowering weight gain, and reducing oviposition (Sõgawa and Pathak, 1970; Horgan, 2009; Senthil-Nathan et al., 2009a,b) BPHs that feed for a long time on resistant rice may gradually evolve into a new biotype to adapt to the resistant plant (Claridge and Hollander, 1980). Among the different biotypes, BPH biotype 1 is unable to infest any resistant rice variety and usually only occurs on the TN1 rice variety (Jena and Kim, 2010). The virulent biotype Y is a biotype that has overcome the resistance of BPH15 by forcing biotype 1 BPH insects to feed on YHY15 for generations (Jing et al., 2012). Secondary metabolites in rice play important roles in various stress responses and inhibit BPH feeding. Hundreds of metabolites in rice were detected by applying widely targeted metabolomics, and two major classes of subspecies-specific metabolites were identified, C-glycosylated flavonoids and phenolamides, which have indispensable roles in chemical defense against biotic and abiotic stresses (Chen et al., 2014). Following feeding by BPH, rice plants synthesize chemicals via the shikimate pathway to deter the insect, including phenolamides (PAs), p-coumaroylputrescine, feruloylputrescine, oxalic acid, apigenin-C-glycosides, and phenolic acids such as vanillic acid, syringic acid, cinnamic acid, and p-coumaric acid (Yoshihara et al., 1980; Stevenson et al., 1996; Rani and Jyothsna, 2010; Alamgir et al., 2016). A clear difference between the resistant BPH15 introgression line and the susceptible recipient line is expression of genes related to secondary metabolites (Lv et al., 2014). Thus, BPHs have overcome these defensive compounds by adapting to the host plant.

Previous research has revealed that insect P450s play a significant role in the metabolism of plant defense compounds. However, there are only a few studies that have investigated the ability of piercing-sucking insects, particularly BPH, to utilize P450s and other enzyme to adapt to resistant plants (Senthil-Nathan et al., 2008). Thus, to enhance knowledge regarding how plant resistance genes have driven the evolution of insect P450s, we used the resistant rice strain YHY15 and a specialist biotype, biotype Y, as a study system. We first compared the detoxification abilities of biotype Y and biotype 1 by evaluating their tolerance to a YHY15 leaf sheath extract. Next, we knocked down BPH CPR (*NlCPR*) and upregulated P450 genes induced by feeding on YHY15. To examine the effect on physiological phenotype and feeding behavior, BPHs with RNA interference (RNAi)-mediated *NlCPR* and P450 gene knockdown were allowed to feed on YHY15. The findings reported herein will contribute to our understanding of the mechanism of interaction between rice and BPHs.

## Materials and methods

### Rice varieties and insects

TN1 is a susceptible rice variety that does not carry BPH-resistance genes. YHY15 harbors the BPH-resistance gene *BPH15*; it was selected by marker-assisted selection from the RI93 × TN1 F2 population [a selected recombinant inbred line (RIL) carrying a single *BPH15* resistance gene] (Yang et al., 2004). Biotypes Y and 1 were full sib-mated for at least 40 generations and maintained on YHY15 and TN1 rice plants, respectively. The insects and rice plants used for all experiments were maintained and/or planted at the Institute of Genetics at Wuhan University in a greenhouse environment controlled at 28 ± 2°C during a 14-h light (06:00–20:00) cycle and 25 ± 2°C during a 10-h dark (20:00–06:00) cycle.

### Leaf sheath ethanol extracts

Rice leaf sheaths were ground into powder in liquid nitrogen. Next, 1 g of powder was added to 10 mL of absolute ethanol (1:10 w:v), extracted for 48 h, and then centrifuged for 15 min at 10,000 × g. The resulting supernatant was concentrated 20-fold using nitrogen flow. The concentrates were added to artificial diets at a volume ratio of 1:10; an ethanol-only artificial diet was used as a control. The artificial diet was changed daily to avoid alcohol-soluble substance precipitation and bacterial growth.

### Artificial diet feeding

The artificial diet solution was prepared as previously described (Stevenson et al., 1996). We used polyvinyl chloride (PVC) plastic pipes with a diameter of 40 cm and a length of 45 cm as the feeding chamber. One end of the pipe was sealed with a Parafilm membrane, and the artificial diet was added onto the Parafilm membrane and then sealed with another Parafilm membrane. BPHs were placed on the Parafilm membrane cover of each feeding chamber for feeding, and the other end was covered with a piece of mesh. The artificial diet devices were placed in the greenhouse. To record the survival rate, five 5th instar nymphs were placed on the artificial diet devices. There were 10 treatments, and each treatment contained two artificial diet devices.

### Total RNA isolation and cDNA synthesis

Total RNA was extracted from the 5th instars or adult female BPHs that had been stored in liquid nitrogen using RNAiso Plus (Takara, Dalian, China). Potential genomic DNA contamination was eliminated by treatment with DNase I **(**Thermo Scientific, Waltham, MA, USA) after RNA extraction. The RNA concentrations and qualities were determined using a Nanodrop spectrophotometer (Thermo Scientific, Waltham, MA, USA). First-strand cDNA was synthesized according to the manufacturer's protocol (Thermo Scientific, Waltham, MA, USA). The cDNA mixture was reverse synthesized from 2 μg total RNA using reverse transcriptase and oligo (dT)_15_ as the primer.

### Quantitative real-time PCR and semi-quantitative RT-PCR

Quantitative real-time PCR (qRT-PCR) was performed using So Advanced SYBR Green Supermix and CFX96 Touch™Real-Time PCR Detection System (BioRad, Laboratories, Hercules, CA, USA) following the manufacturer's instructions. The results were analyzed using CFX Manager Software 2.1; *actin 1* (accession number: EU179846.1) and *GAPDH* (accession number: KU365927.1) were used as internal controls to standardize the results according to sequencing data. All results were obtained from three independent biological replicates and three technical replicates. Semi-quantitative RT-PCR (sqPCR) was performed using the following thermal program: initial denaturation at 94°C for 3 min, 40 cycles of 94°C for 30 s, 55°C for 30 s and 72°C for 30 s, and a final extension period at 72°C for 5 min. The *act in 1* and *GAPDH* genes were amplified for 25 cycles for sample normalization. To screen for BPH-upregulated *P450* genes induced by YHY15, qRT-PCR primer sequences obtained from Bass et al. (2011) were used and named according to Lao et al. (2015).

### dsRNA synthesis and injection

We synthesized dsRNA based on cloned *NlCPR* (Liu et al., 2015) and *CYP4C61* (GenBank: FM163384.1) sequences. PCR products, 581 bp for *NlCPR*, 540 bp for *CYP4C61*, 561 bp for *CYP6AX1*, 583 bp for *GFP* and 507 bp for *CYP6AY1*, were used as templates for dsRNA synthesis using the MEGAscript T7 transcription kit (Ambion, Austin, TX, USA). The five pairs of primers used for dsRNA synthesis are listed in Table S3. For dsRNA injection, nearly emerged female 5th instar nymphs were first anesthetized with carbon dioxide for 20 s, and approximately 150 ng dsRNA was injected using a Nanoject II Auto-Nanoliter Injector (Drummond Scientific, Broomall, PA, USA). After injection, the awakened BPHs were prepared for the next experiments. In the *NlCPR* weight gain study, a group of 20 BPHs was injected with *NlCPR* dsRNA as a treatment, and at the same time, 20 BPHs were injected with *GFP* dsRNA to serve as a control. The experiment was repeated five times. For the honeydew excretion assay, the injection procedure followed the weight gain assay, except the experiment was repeated eight times. For the artificial diet feeding assay, a group of 10 BPHs was injected with *NlCPR* dsRNA as a treatment and another 10 BPHs were injected with *GFP* dsRNA as a control. The experiment was repeated five times. The weight gain assay for *CYP4C61, CYP6AX1*, and *CYP6AY1*, was the same procedures as used in the *NlCPR* weight gain assay. The same honeydew excretion and artificial diet feeding assay procedures used for *NlCPR* were also used for *CYP4C61*.

### Evaluation of BPH weight change and honeydew excretion

Parafilm membranes were cut and folded to form a bag 3 cm in length and 5 cm in width. The bags were then fixed to the stem of rice seedlings at a position 2 cm above the soil, leaving a small opening to allow for the placement of BPHs. For the honeydew excretion experiment, BPHs were starved for 2 h prior to being placed on the filter paper. Two similarly sized dsRNA-treated nearly emerged female 5th instar nymphs were placed in each bag, and the bags were then sealed. After the BPHs had fed on YHY15 for 48 h, the bag was removed, and honeydew of emerged female adult was collected and weighed using a microbalance.

The honeydew stain assay was performed according to a previously described protocol (Du et al., 2009), with slight modifications. Briefly, one starved BPH was placed in a filter paper chamber. After 2 days, the filter paper was treated with 0.1% ninhydrin in acetone solution and dried for 30 min at 60°C until honeydew stains appeared.

To quantify changes in body weight, dsRNA-treated nearly emerged female 5th instar nymphs were selected and measured using a microbalance. The insects were then placed on a 4-week-old YHY15 or TN1 plant. After 96 h, each individual was weighed again. BPH weight gain was calculated as the proportional change in weight relative to the initial weight.

### Electrical penetration graph recording

The electrical penetration graph (EPG) experiment was performed using a Giga-8 DC EPG amplifier (Wageningen Agricultural University, Wageningen, The Netherlands). Before injecting dsRNA, the biotype Y BPHs were fed only water on filter paper for 2 h. The dsRNA-treated BPHs were anesthetized with CO_2_ for 20 s. To prepare insect electrodes, one end of a gold wire was connected to the amplifier through the EPG probe, and the other end was attached to a BPH using water-soluble silver conductive glue (Wageningen Agricultural University, Wageningen, The Netherlands); the insect was then placed on a rice plant. The plant electrode was designed by inserting a copper wire (2 mm in diameter and 10 cm in length) into the soil surrounding one rice plant. The EPG recordings were conducted in a Faraday cage with the gain of the amplifier set at 50 × and the output voltage adjusted between ±5 V. All EPG experiments were recorded for 3 h and performed at the above-described BPH feeding temperature and humidity conditions. The EPG data were analyzed with PROBE 3.4 (Wageningen Agricultural University, Wageningen, The Netherlands).

### Tissue preparation

Before dissection, insects were chilled on ice and placed in a Petri dish that had been brushed with chilled insect physiological buffer (0.65% NaCl water solution). The midguts, salivary glands and fat bodies were dissected from 100 individuals collected for total RNA extraction as one biological replicate. The dissected midguts were immediately placed in RNAiso Plus. Three biological replicates were conducted in this experiment.

### Gas chromatography–mass spectrometry (GC–MS) analysis

For identification of honeydew metabolites, honeydew was collected with a micropipette after the BPHs had fed on YHY15 for 24 h, transferred to a centrifuge tube and maintained on ice. There were five biological replicates for each treatment feeding on YHY15. Total 15 honeydew samples were used.

For honeydew metabolite analysis, metabolite derivatization and GC–MS were performed according to a previously described protocol (Peng et al., 2016). Briefly, honeydew supernatants were obtained by centrifugation at 10,000 × *g* for 5 min, and 10 μL of the supernatant was used for GC–MS detection, with 1 μL of ribitol (0.2 mg/mL aqueous solution) used as the internal standard. The supernatants were dried by nitrogen flow in preparation for the next step, derivatization. The dried residue was re-dissolved in 40 μL methoxyamination reagent (methoxy-amino-hydrochloride, 20 mg/mL solution of pyridine) and derivatized with 70 μL MSTFA (N-methyl-N-(trimethylsilyl) trifluoroacetamide) at 37°C for 30 min. The derivatized sample was then transferred to a linear tube suitable for GC–MS analysis. All derivatized reagents were purchased from Sigma-Aldrich (Shanghai, China).

The derivatized sample was analyzed by GC–MS (Thermo Trace GC Ultra-ISQ, Thermo Fisher Scientific, USA) with an Rtx-5 MS capillary column (30 m × 0.25 mm). Helium was used as the carrier gas at a flow rate of 1 mL/min; the initial oven temperature of the column was held at 100°C for 3 min, ramped to 280°C at 5°C/min, and then held for 5 min. The sample size was maintained at 1 μL with an AS-3000 autosampler and set for splitless injection; the injection temperature was maintained at 250°C. The mass spectrometer was calibrated according to the manufacturer's recommendations using tris-(perfluorobutyl)-amine (CF43). An electro-impact (EI) mode of 70 eV was used for ionization. The recorded mass range was from 50 to 650 m/z.

To detect compounds in the leaf sheath extracts, 500 μL of leaf sheath extract solution was dried with a flow of nitrogen and then dissolved in 100 μL acetone to prepare for GC**–**MS analysis. The GC**–**MS conditions were identical to those mentioned above except that the column initial oven temperature was held at 60°C for 5 min, ramped to 280°C at 10°C/min, and then held for 5 min.

### Sequence analysis

Four *CYP4C61* sequences, biotype 1 *CYP4C61*, biotype Y *CYP4C61, CYP4C61*v1 (GenBank: FM163384.1), and *CYP4C61*v2 (GenBank: KM217037.1) were aligned using BioEdit software (http://www.mbio.ncsu.edu/BioEdit/bioedit.html). SignalP (http://www.cbs.dtu.dk/services/SignalP/) and PSIPRED (http://bioinf.cs.ucl.ac.uk/psipred/) were used to predict the signal peptide and secondary structure of *CYP4C61*, respectively. Substrate recognition sites (SRSs) were analyzed according to the secondary structure and schematic topology of P450 (Raucy and Allen, 2001; Rewitz et al., 2006; Rani and Jyothsna, 2010).

### Statistical analysis

Multivariate statistics of partial further square-discrimination analysis (PLS-DA) was performed using SIMCA-P software (V11.0, Umetrics, Umeå, Sweden). Two-way analysis of variance (ANOVA) was carried out to examine the main and interactive effects of ethanol extract feeding, feeding time and biotype on survival rates. One-way ANOVA with a *post-hoc* Tukey test was used for comparisons between groups. The *t*-test was used for comparisons between two groups. The *t*-test, two-way ANOVA and one-way ANOVA with a *post-hoc* Tukey test were performed with SPSS17.0 software (SPSS Inc., Chicago, IL, USA).

## Results

### BPH feeding fitness on leaf sheath ethanol extracts

**Figure 1:**
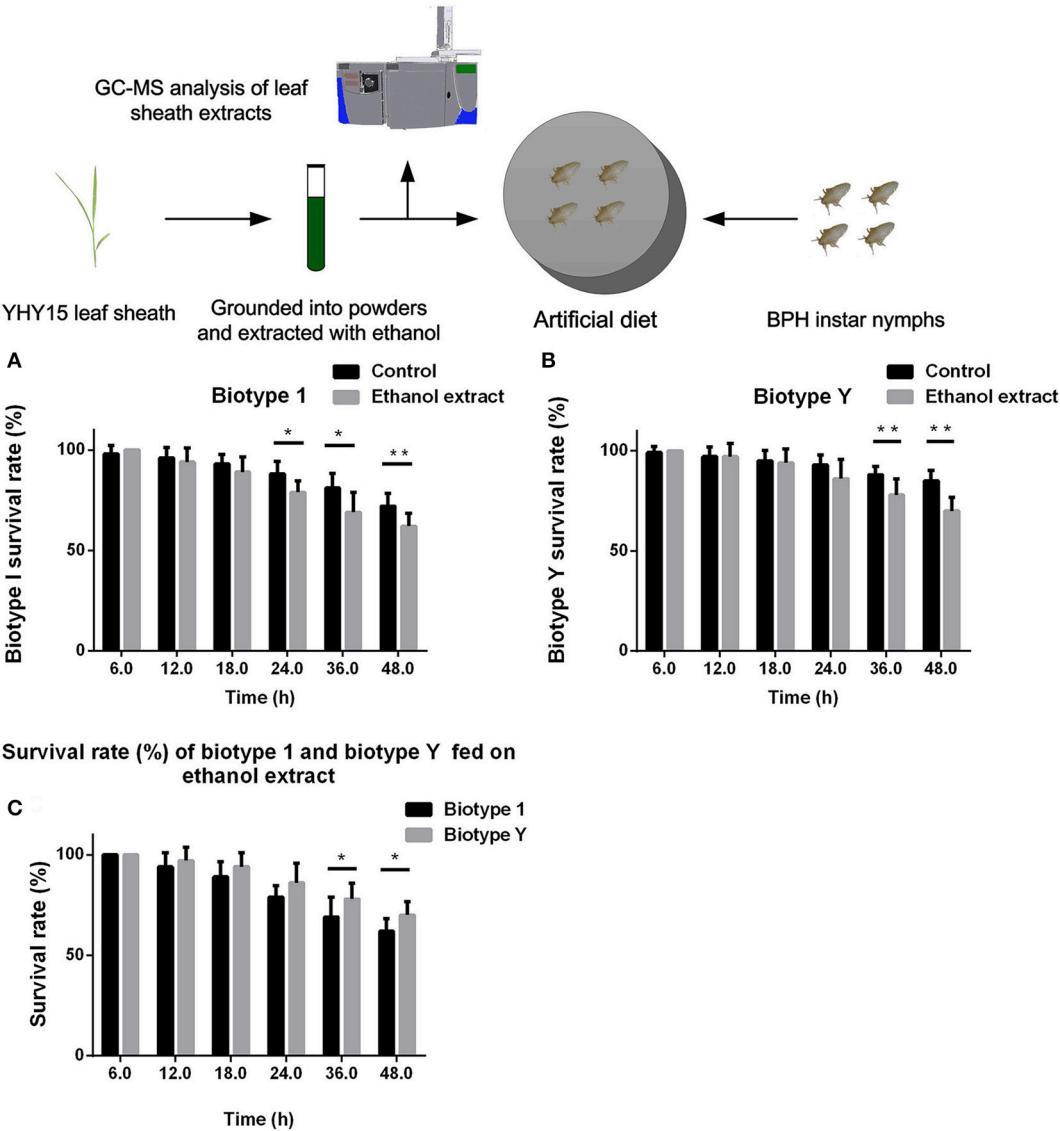
Survival rate of BPH feeding on artificial diets containing 10% ethanol extracts. **(A)** Biotype 1 BPH was fed artificial diets containing 10% ethanol extracts; an artificial diet with 10% ethanol served as the control. The BPH survival rate was determined at 6–48 h post-feeding. **(B)** Biotype Y BPHs fed artificial diets containing ethanol extracts and artificial diets with ethanol added as a control. The BPH survival rate was determined at 6–48 h post-feeding. **(C)** Comparison of the survival rates of biotype 1 BPHs with biotype Y BPHs fed artificial diets containing ethanol extracts. ^*^, ^**^ on the bars indicate significance at *P* < 0.05 and *P* < 0.01 (*t*-test), respectively.

We compared the abilities of biotypes 1 and Y to tolerate YHY15 leaf sheath ethanol extracts. The two biotypes of BPH nymphs were fed with artificial diets that contained 10% ethanol extracts (volume fraction), and the survival rates were recorded 6, 12, 18, 24, 36, and 48 h after feeding (Figure 1). Compared to the control group (i.e., diet without ethanol extract), BPHs of biotypes 1 and Y had a higher mortality rate when fed diets containing ethanol extracts; biotype 1 reached a significant level from 24 to 48 h (*t*-test, *P* = 0.011 for 24 h; *P* = 0.024 for 36 h; *P* = 0.004 for 48 h; Figure 1A), and biotype Y reached a significant level from 36 to 48 h (*t*-test, *P* = 0.0085 for 36 h; *P* = 0.0003 for 48 h; Figure 1B). Two-way ANOVA results showed that feeding on ethanol extracts had significant negative effects on survival rates of both biotype 1 (*df* = 1, *P* < 0.0001, *F* = 25.70) and biotype Y (*df* = 1, *P* < 0.0001, *F* = 24.13). Feeding time also had significant negative effects on survival rates of both biotype 1 (*df* = 5, *P* < 0.0001, *F* = 76.35) and biotype Y (*df* = 5, *P* < 0.0001, *F* = 41.26). The feeding ethanol extract × feeding time interaction significantly affected the survival rate of biotype 1 (*df* = 1, *P* = 0.004, *F* = 3.65) and biotype Y (*df* = 5, *P* < 0.0001, *F* = 5.81).

These results showed that the ethanol extracts contained substances that were toxic to BPHs and decreased their survival rate. When comparing the mortality rates of biotype Y with biotype 1 after feeding on diets containing ethanol extracts, biotype 1 insects had a higher mortality compared with those of biotype Y (*t*-test, *P* = 0.038 for 36 h; *P* = 0.013 for 48 h), with biotype 1 reaching a significant level from 36 to 48 h (Figure 1C). In addition, two-way ANOVA showed that when feeding on ethanol extracts, biotype had a significant effect on the survival rate (*df* = 1, *P* < 0.0001, *F* = 18.00); conversely, the biotype × feeding time interaction did not significantly affect the survival rate (*df* = 5, *P* = 0.310, *F* = 1.21). Overall, biotype Y had a higher survival rate, indicating that this biotype has stronger biological detoxification capacity than biotype 1.

We used GC–MS to analyze underivatized compounds from the YHY15 leaf sheath ethanol extracts; identification of the phytochemical compounds in the YHY15 leaf sheath ethanol extracts was based on National Institute of Standards (NIST) library searches. Figure S3A shows typical total ion chromatograms (TIC) of the YHY15 leaf sheath ethanol extracts. Twenty-four compounds were identified (Table S1), most of which are secondary metabolites, including 2-pentanone, 4-hydroxy-4-methyl-, benzeneethanamine, N-(1-methylethylidene), 1-(3,6,6-trimethyl-1,6,7,7a-tetrahydrocyclopenta[c]pyran-1-yl)ethanone, butylated hydroxytoluene, 4-((1E)-3-hydroxy-1-propenyl)-2-methoxyphenol, 7,9-di-tert-butyl-1-oxaspiro(4,5)deca-6,9-diene-2,8-dione, campesterol, stigmasterol, and β-sitosterol. These compounds may be toxic to BPHs.

### NADPH–cytochrome P450 reductase knockdown affects the ability of biotype Y BPH to feed on YHY15

The above results show that toxic substances affect BPH survival rates, and it has been reported that P450 activity is involved in detoxification. Thus, we attempted to evaluate the role of the BPH P450 system in the interaction between BPH and resistant rice. CPR is essential for cytochrome P450 activity, and the reductase is capable of supplying electrons to each of the different P450 enzymes.

**Figure 2:**
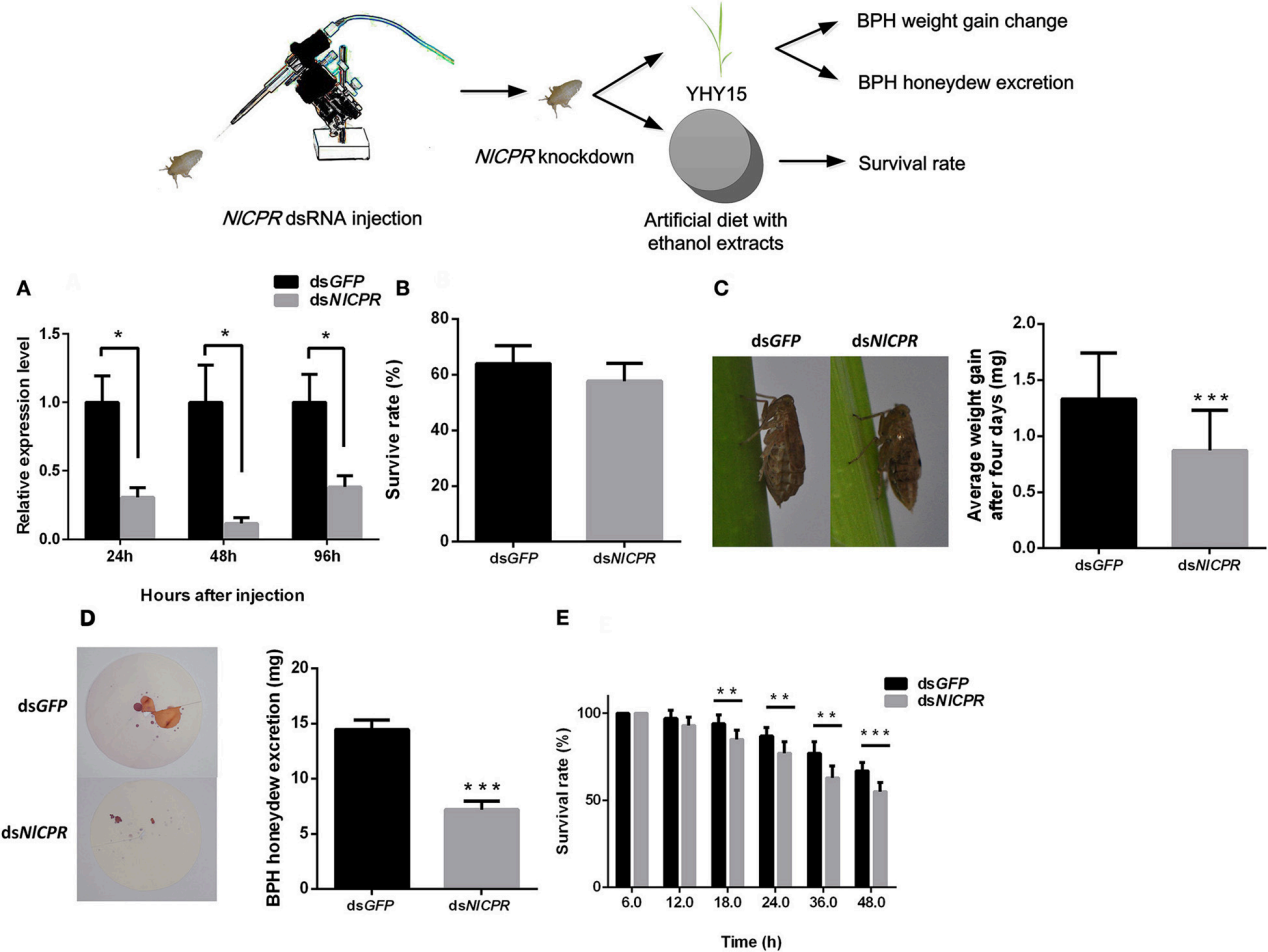
Phenotype resulting from biotype Y BPH *NlCPR* knockdown. **(A)** qRT-PCR analysis of the effects of *NlCPR* knockdown at different time points. **(B)** Survival rates of biotype Y BPHs treated with *NlCPR* and *GFP* dsRNA. *GFP* dsRNA-treated BPH served as the control. **(C)** Weight gain of dsRNA-treated BPHs. **(D)** Honeydew excretion by dsRNA-treated BPHs. **(E)** Survival rates of BPHs fed artificial diets containing 10% ethanol extracts. ^*^, ^**^, ^***^ on the bars indicate significance at *P* < 0.05, *P* < 0.01, and *P* < 0.0001 (*t*-test), respectively.

To investigate the effects of dsRNA against NlCPR in BPHs feeding on the resistant rice strain YHY15, we injected *NlCPR* dsRNA into biotype Y BPHs. qRT-PCR was used to confirm the RNAi effect of target genes (Figure 2A). The results showed that after injection with *NlCPR* dsRNA, transcripts of *NlCPR* were downregulated 69.5, 88.5, and 61.8% at 24, 48, and 96 h, respectively, compared to the controls (*t*-test, *P* = 0.011 for 24 h; *P* = 0.013 for 48 h; *P* = 0.022 for 96 h). During a 4-day period of rearing on YHY15, there were no significant differences (*t*-test, *P* = 0.061) in the average survival rates between the *NlCPR* dsRNA-pre-treated BPHs (57.7 ± 6.4%) and the controls (64.0 ± 8.5%; Figure 2B).

When feeding on YHY15, the average weight gain of the *NlCPR* dsRNA-pre-treated BPHs (mean ± SEM) was 0.87 ± 0.36 mg, and the average weight gain of the control BPHs, i.e., *GFP* dsRNA-pre-treated BPHs, was 1.33 ± 0.41 mg (Figure 2C). Overall, the average weight gain of the *NlCPR* dsRNA-pre-treated BPHs was significantly lower than that of the control (*t*-test, *P* < 0.0001). No significant difference (*t*-test, *P* = 0.056) was detected between the *NlCPR* dsRNA-pre-treated BPH and the controls when feeding on TN1 rice (Figure S1A).

The amount of honeydew excreted from the *NlCPR* dsRNA-pre-treated BPHs was 7.19 ± 0.76 mg (mean ± SEM) after 48 h, and that of the controls was 14.45 ± 0.86 mg (Figure 2D). The honeydew excretion assay showed that honeydew excretion by the *NlCPR* dsRNA-pre-treated BPHs was significantly lower than that of the controls (*t*-test, *P* < 0.0001). Moreover, the survival rates of the *NlCPR* dsRNA-pre-treated BPHs feeding on artificial diets containing 10% ethanol extracts were lower than those of the control at 18 h post-BPH feeding and thereafter (*t*-test, *P* = 0.0012 for 18 h; *P* = 0.0013 for 24 h, *P* = 0.0002 for 36 h; *P* < 0.0001 for 48 h; Figure 2E). These results show that knocking down the *NlCPR* gene to decrease the activity of the P450s decreases the ability of biotype Y to adapt to YHY15 but does affect the feeding of this biotype on TN1.

We classified the signals of EPG into six different waveforms according to the features of typical waves: Np, N1, N2, N3, N4, and N5. BPH feeding behavior after *NlCPR* knockdown was investigated. Regarding the EPG signal waveform of BPHs injected with *NlCPR* dsRNA for 0, 12, 24, 48, and 72 h, the average feeding waveforms of xylem and phloem of N4 and N5 accounted for 86.7, 56.77, 44.55, 37.46, and 56.65% (Table 1, Figure **6A**), respectively. As shown in Table 1, the Tukey test demonstrated that significantly different results were obtained for treatment at 0 h compared with treatment at 12, 24, 48, and 72 h; the feeding waveform was the lowest at 48 h, and this waveform was significantly different compared with that 0, 12, and 72 h waveforms. Corresponding with the qRT-PCR results of examining the *NlCPR* RNAi effect, the feeding waveform was significantly decreased when *NlCPR* was knocked down.

**Table 1:** Comparison of different EPG waveform feeding patterns of *NlCPR* and *CYP4C61* dsRNA-treated BPH at different time points while feeding on YHY15 plants for 3 h (percentage duration and standard error).

	***n***	**Np**	**N1+N2**	**N3**	**N4**	**N5**	**Np+N1+N2+N3**	**N4+N5**
**ds*****NlCPR*** **(h)**								
0	4	2.48 ± 2.41a	7.5 ± 1.36c	3.33 ± 0.53b	75.65 ± 11.55a	11.05 ± 12.29a	13.3 ± 2.14c	86.7 ± 2.14a
12	4	7.1 ± 3.17a	25.27 ± 8.89ab	10.87 ± 4.21ab	54.1 ± 6.26b	2.67 ± 2.31a	43.23 ± 4.25b	56.77 ± 4.25b
24	6	4.85 ± 5.6a	36.17 ± 6.54a	14.43 ± 5.57a	43.12 ± 3.14bc	1.43 ± 2.14a	55.45 ± 3.36ab	44.55 ± 3.71bc
48	5	8.12 ± 6.86a	39.5 ± 9.12a	14.92 ± 6.18a	30.92 ± 10.9c	6.54 ± 11.01a	62.54 ± 8.05a	37.46 ± 8.05c
72	4	4.28 ± 3.51a	22.43 ± 2.36b	16.65 ± 4.46a	53.38 ± 5.7b	3.28 ± 2.48a	43.35 ± 7.16b	56.65 ± 7.6b
**ds*****CYP4C61*** **(h)**								
0	4	2.65 ± 1.32a	6.38 ± 2.17b	4.78 ± 2.21a	82.9 ± 1.94a	3.3 ± 4.05a	13.8 ± 4.7c	86.2 ± 4.7a
12	3	2.70 ± 0.87a	27.87 ± 6.73a	5.63 ± 4.96a	60.2 ± 1.4b	3.6 ± 2.69a	36.2 ± 1.32b	63.8 ± 1.32b
24	4	7.45 ± 5.3a	39.28 ± 9.04a	17.1 ± 6.19a	34.25 ± 3.42c	1.93 ± 2.23a	63.83 ± 3.86a	36.17 ± 3.86c
48	4	17.68 ± 12.56a	26.75 ± 6.7a	15.25 ± 9.36a	41.3 ± 4.23c	1.53 ± 1.8a	57.18 ± 3.63a	42.82 ± 3.63c
72	3	6.9 ± 1.85a	26.3 ± 13.46a	22.67 ± 21.84a	43.37 ± 11.28c	0.77 ± 1.33a	55.87 ± 12.57a	44.13 ± 12.57c

*Means ± SE within columns followed by the same letters are not significantly different (Tukey test, P > 0.05)*.

### Screening of BPH-upregulated P450 genes induced by YHY15

**Figure 3:**
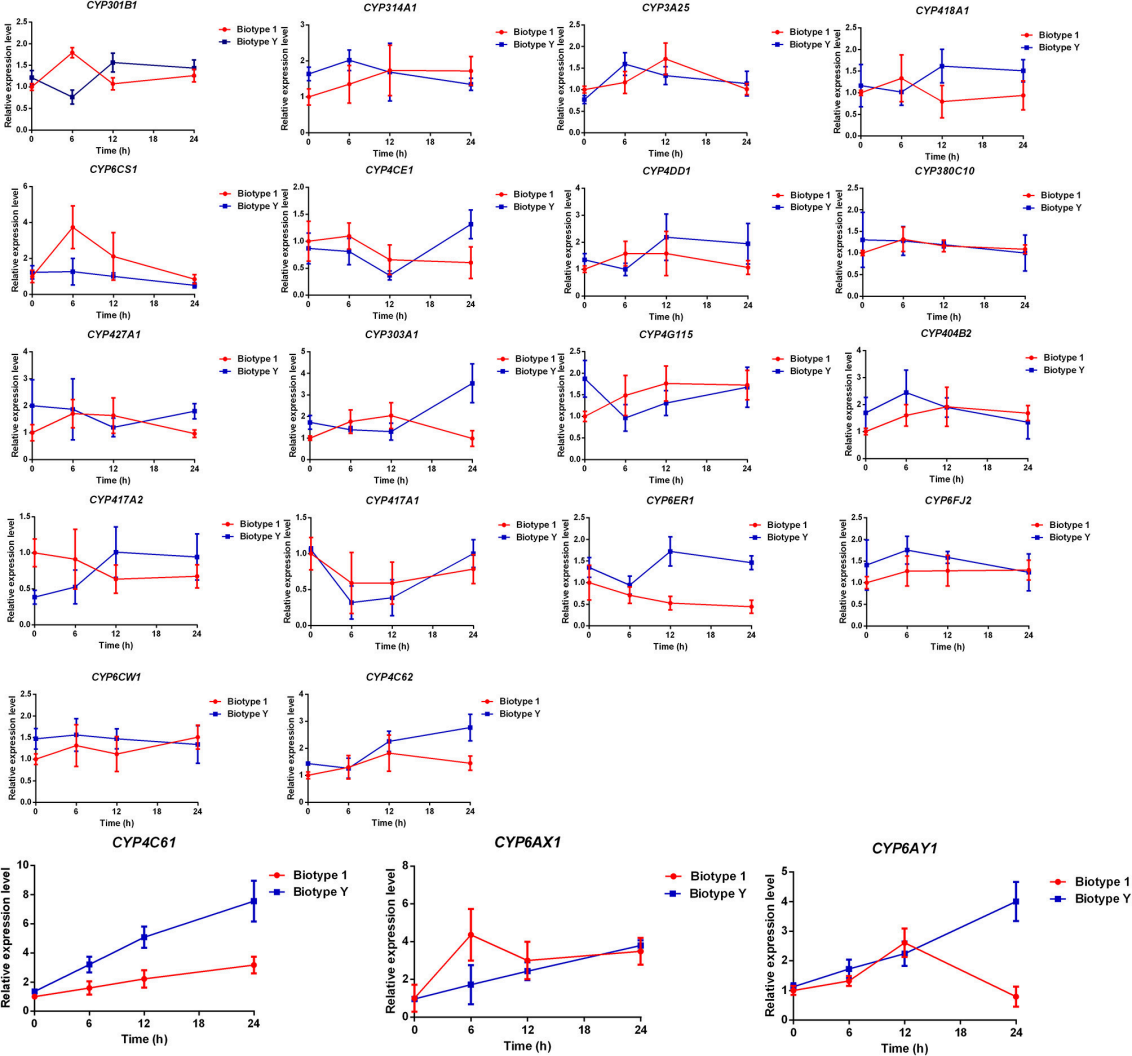
qRT-PCR analysis of P450 mRNA levels. Time course of P450 enzyme expression in 5th instar nymphs of biotype 1 and biotype Y BPHs feeding on YHY15. Error bars represent means ± SEM. Essentially identical results were obtained in three independent experiments.

To evaluate the BPH P450 gene expression pattern in response to resistant YHY15 rice, a generation of biotype Y BPHs was reared on the susceptible cultivar TN1; biotype 1 BPHs were consistently reared on TN1. At the 5th instar nymph stage, same size BPHs were selected for feeding on YHY15 for 0 (i.e., control), 6, 12, and 24 h, and samples were collected for extraction of total RNA for cDNA synthesis. The expression pattern of BPH *P450* genes after feeding on YHY15 at 0, 6, 12, and 24 h induced by the BPH resistance gene *BPH15* was investigated by qRT-PCR (Figure 3). A total of 21 primer pairs for different BPH *P450* genes were selected for this experiment (Table S3). The expression levels of the 21 *P450* genes, *CYP4C61, CYP6AX1*, and *CYP6AY1* in both biotypes increased continuously from 0 to 24 h. In biotype 1, *CYP4C61, CYP6AX1*, and *CYP6AY1* expression levels were upregulated by 3.17-, 3.49-, and 0.79-fold, respectively, at 24 h compared to those at 0 h, and those in biotype Y were upregulated by 7.55-, 3.94-, and 3.55-fold, respectively. These results indicate that these three *P450* genes were induced by YHY15.

### *CYP4C61* knockdown affects BPH feeding on YHY15

**Figure 4:**
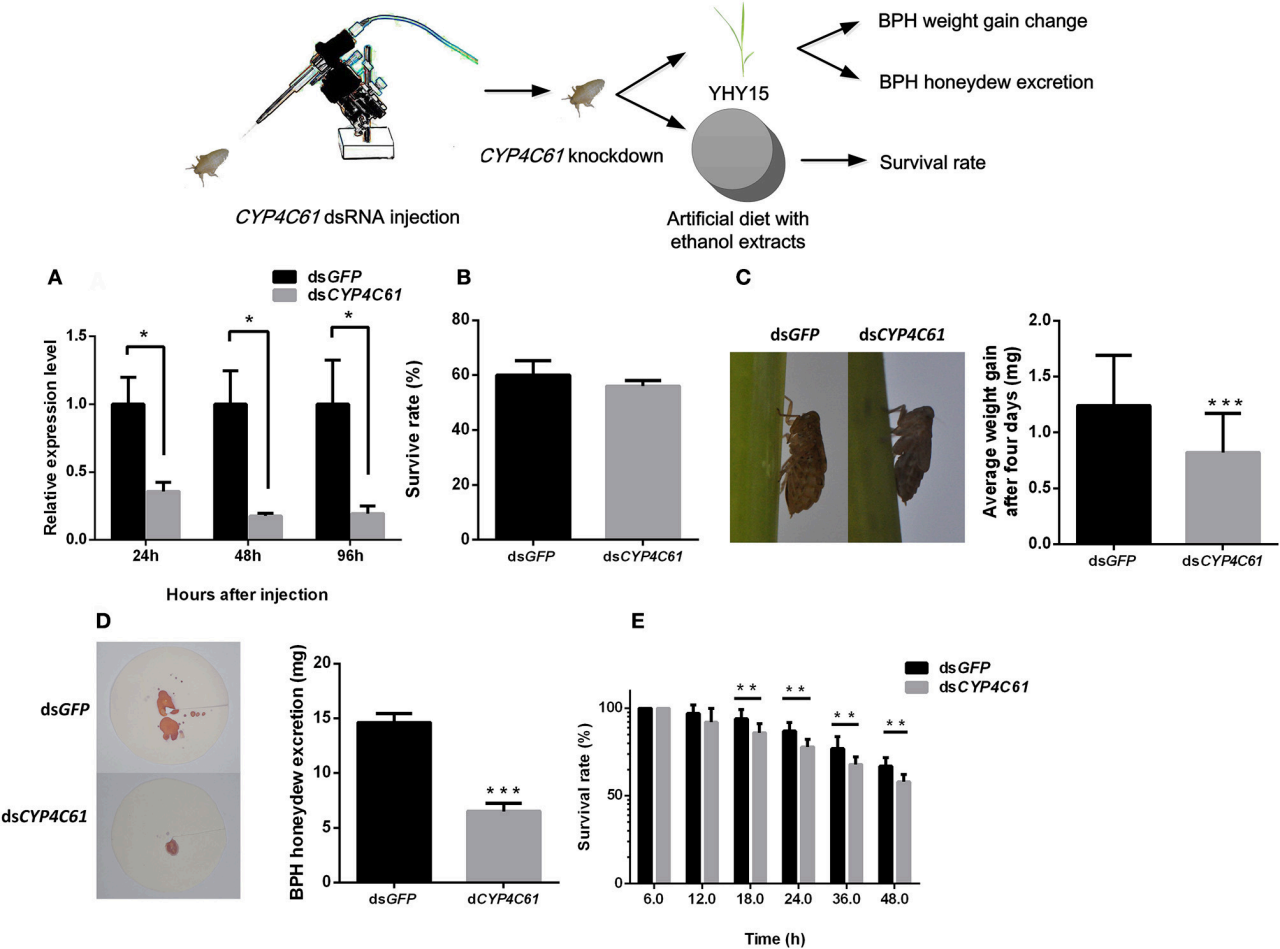
Phenotype resulting from biotype Y BPH *CYP4C61* knockdown. **(A)** qRT-PCR analysis of the effects of *CYP4C61* knockdown at different time points. **(B)** Survival rates of biotype Y BPHs treated with *CYP4C61* and *GFP* dsRNA. *GFP* dsRNA-treated BPH served as the control. **(C)** Weight gain of dsRNA-treated BPH. **(D)** Honeydew excretion by dsRNA-treated BPHs. **(E)** Survival rates of BPH fed artificial diets containing 10% ethanol extracts. ^*^, ^**^, ^***^ on the bars indicate significance at *P* < 0.05, *P* < 0.01and *P* < 0.0001 (*t*-test), respectively.

To verify whether *CYP4C61, CYP6AX1*, and *CYP6AY1* knockdown affects the normal physiological activity of biotype Y BPHs, we injected *CYP4C61, CYP6AX1*, and *CYP6AY1* dsRNA into these insects and performed qRT-PCR (Figure 4A). The results indicated decreased *CYP4C61* expression levels at 24, 48, and 96 h compared to those at 0 h (*t*-test, *P* = 0.015 for 24 h; *P* = 0.011 for 48 h; *P* = 0.029 for 96 h). *CYP6AX1* and *CYP6AY1* expression levels also were decreased at 96 h post-feeding compared to those at 0 h (Figure S2B).

No statistically significant (*t*-test, *P* = 0.058) difference in survival rate was observed between the *CYP4C61, CYP6AX1*, and *CYP6AY1* dsRNA treatment groups and the control group (Figure 4B, Figure S2A). During a 4-day period of rearing on YHY15, the average weight gain of *CYP4C61* dsRNA-pre-treated BPHs was 0.82 ± 0.32 mg (mean ± SEM), which was significantly (*t*-test, P < 0.0001) lower than that of the control [*GFP* dsRNA (1.24 ± 0.45 mg); Figure 4C]. However, weight gain of BPH nymphs injected with *CYP6AX1* and *CYP6AY1* dsRNA was not significantly (*t*-test, *P* = 0.168 and 0.062, respectively) altered compared to that of the control (Figure S2C). These results indicate that only *CYP4C61* knockdown affects the ability of biotype Y BPHs to feed on YHY15. No significant difference (*t*-test, *P* = 0.061) was detected between the *CYP4C61* dsRNA-pre-treated BPH and the controls when feeding on TN1 rice (Figure S1B).

The amount of honeydew excretion by *CYP4C61* dsRNA-pre-treated BPHs was lower (mean ± SEM, 6.49 ± 0.75 mg) than that of the control (14.63 ± 0.80 mg) at 48 h (*t*-test, *P* < 0.0001; Figure 4D). We also assessed survival rates after *CYP4C61* knockdown in BPHs feeding on an artificial diet containing 10% ethanol extracts (Figure 4E), and the result revealed lower survival rates compared with the control from 18 to 48 h post-BPH feeding (*t*-test, *P* = 0.0028 for 18 h; *P* = 0.0003 for 24 h; *P* = 0.0065 for 36 h; *P* = 0.0003 for 48 h).

Regarding the EPG signal waveform of BPHs injected with *CYP4C61* dsRNA for 0, 12, 24, 48, and 72 h, the average feeding waveforms of xylem and phloem of N4 and N5 accounted for 86.2, 63.8, 36.17, 42.82, and 44.13% (Table 1, Figure 6B), respectively. As shown in Table 1, the Tukey test indicated significant difference at 0 h compared with 12, 24, 48, and 72 h; the feeding waveform was lowest at 24 h and was significantly different compared with the 0-h and 12-h waveforms. The feeding waveform decreased when *CYP4C61* transcript levels were reduced.

### Tissue expression of *CYP4C61*

**Figure 5:**
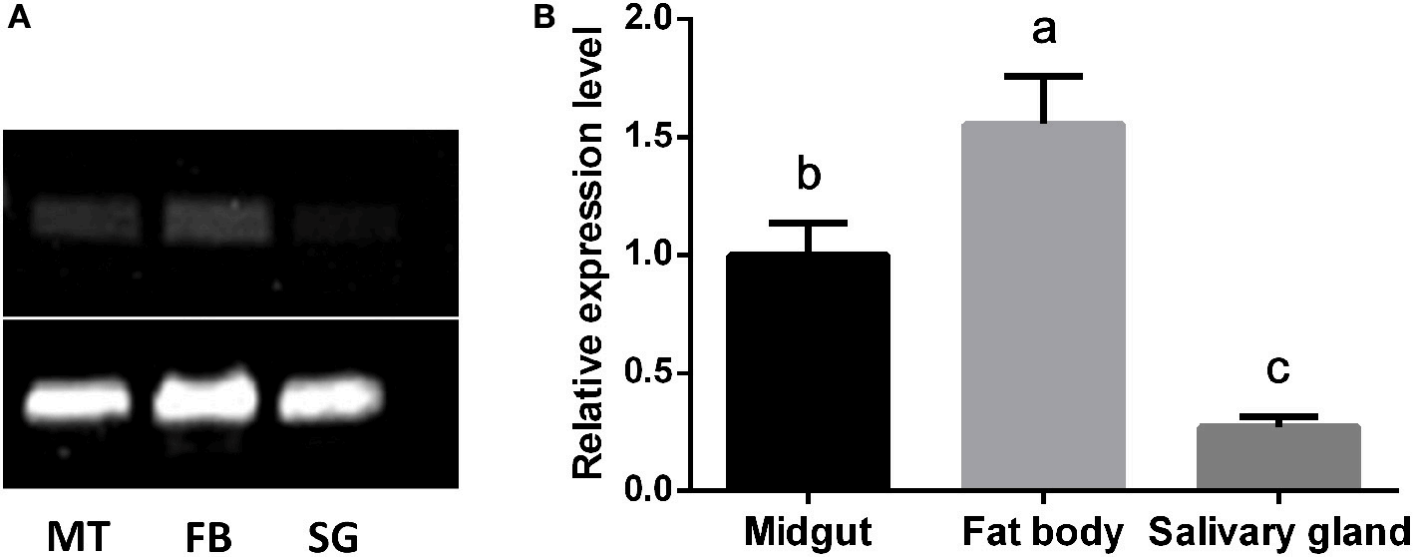
Tissue distributions of *CYP4C61* mRNA in BPH. The experiment was repeated three times with comparable results each time. *Actin 1* was used as a loading control. **(A)** sqPCR analysis of the expression patterns of *CYP4C61* in different tissues. MT, Midgut; FB, fat body; SG, silk gland. **(B)** Real-time qRT-PCR analysis of expression patterns of *CYP4C61* in different tissues. Different letters above the bars indicate significant differences among treatments by the Tukey test: *P* < 0.05, lowercase letters.

The mRNA expression patterns of the *CYP4C61* gene in various tissues of 5th instar larvae were determined by sqPCR (Figure 5A) and qRT-PCR (Figure 5B). Our results showed expression of the *CYP4C61* gene in all tissues examined. The highest transcript levels of *CYP4C61* were detected in the fat bodies, followed by the midgut. The silk gland expressed the lowest *CYP4C61* levels. These results indicate that *CYP4C61* mRNA predominately accumulates in the fat bodies of BPHs.

**Figure 6:**
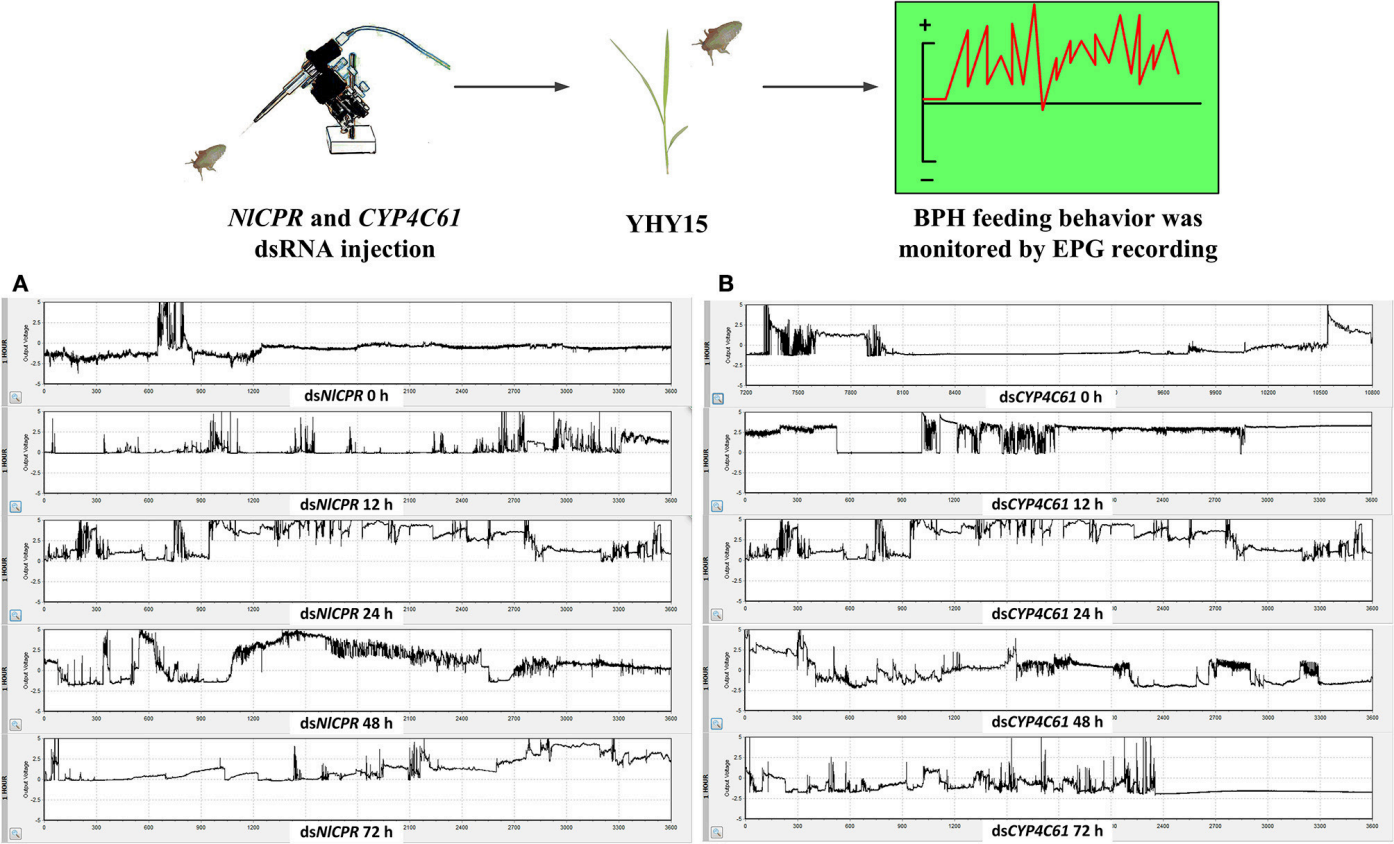
Overall typical EPG waveforms for *NlCPR* and *CYP4C61* dsRNA*-*treated BPH on YHY15 plants. **(A)** EPG waveforms of *NlCPR* dsRNA-treated BPH for 0, 12, 24, 48, and 72 h. **(B)** EPG waveforms of *CYP4C61* dsRNA-treated BPH for 0 h, 12, 24, 48, and 72 h.

### Comparison of the deduced CYP4C61 amino acid sequences between biotypes 1 and Y

**Figure 7:**
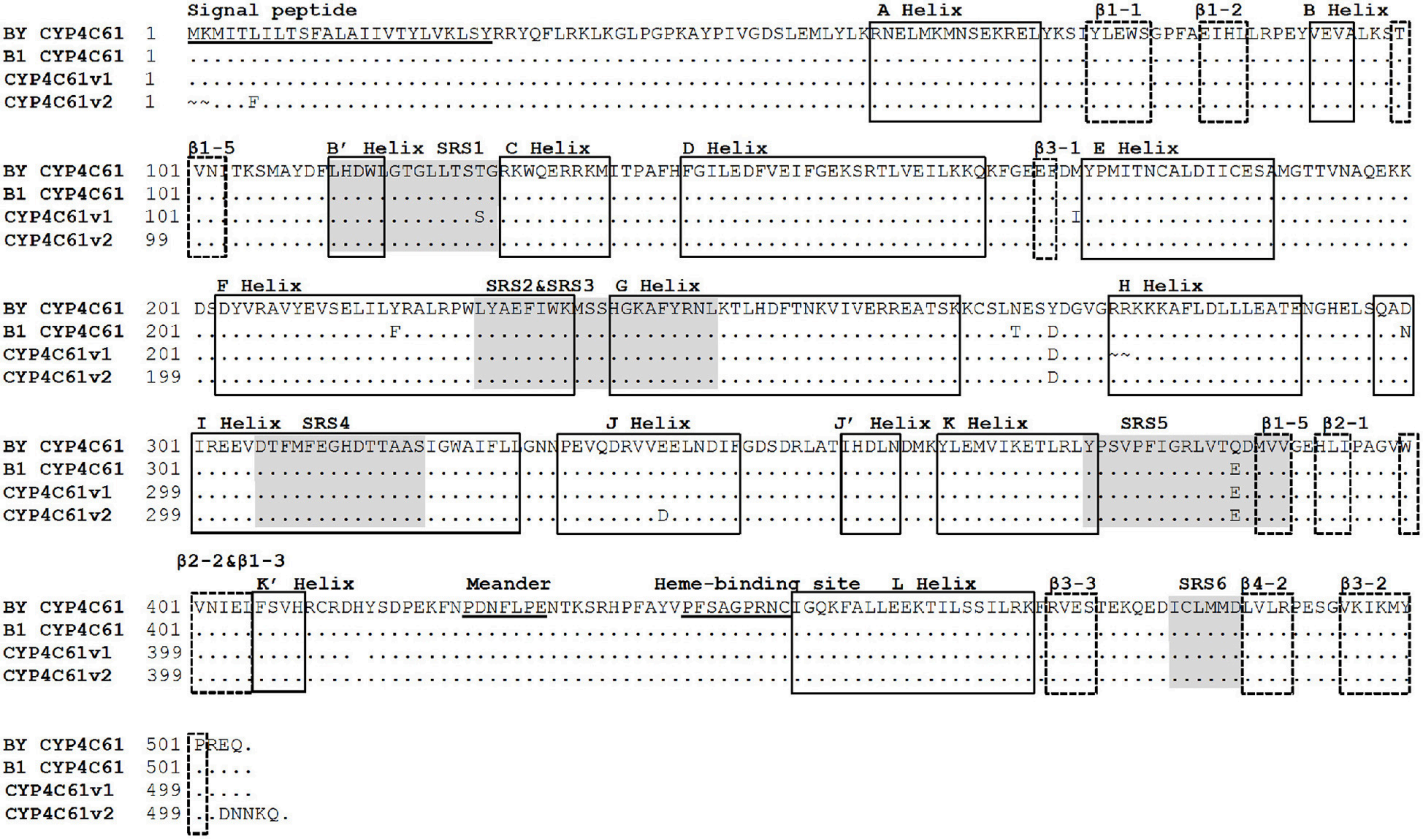
Alignment of amino acid sequences of the biotype Y CYP4C61 protein and other BPH CYP4C61 proteins. BY CYP4C61: Biotype Y CYP4C61; B1 Biotype 1 CYP4C61; CYP4C61v1: amino acid sequences from GenBank: FM163384.1; CYP4C61v2: amino acid sequences from GenBank: KM217037.1. The underlined sequences represent the signal peptide, meander and heme-binding regions. The shaded areas indicate the six substrate recognition sites (SRSs); α-helices and β-sheets are indicated by solid line boxes and broken line boxes, respectively.

Six approximate SRS regions were predicted based on the secondary structure elements and schematic topology of P450s (Figure 7). These enzymes share several conserved motifs, including the P450 heme-binding signature (FXXGXXXCXG), the typical aromatic motif FXPXRF (meander), coinciding with the K-L loop, the EXXR in the K helix and the WXXXR motif in the C helix. Amino acid sequence analysis of CYP4C61 showed that it contains these conserved motifs, including the heme-binding motif (PFXXGXRXCXG), the WXXXR motif in the C helix and the EXXR motif in the K helix. A total of four deduced amino acid differences were found between biotypes 1 and Y, located at amino acid positions 268, 271, 300, and 384. Comparing the amino acid sequences of biotype 1 with the National Center for Biotechnology Information (NCBI) CYP4C61 sequence, the variable amino acids of biotype Y are located at residues 271 and 386, of which residue 386 is located in SRS5. In addition, many nucleotide sequence variants were found between biotypes 1 and Y (Figure S4).

### Metabolomic analysis of honeydew metabolites of dsRNA-treated BPH

**Figure 8:**
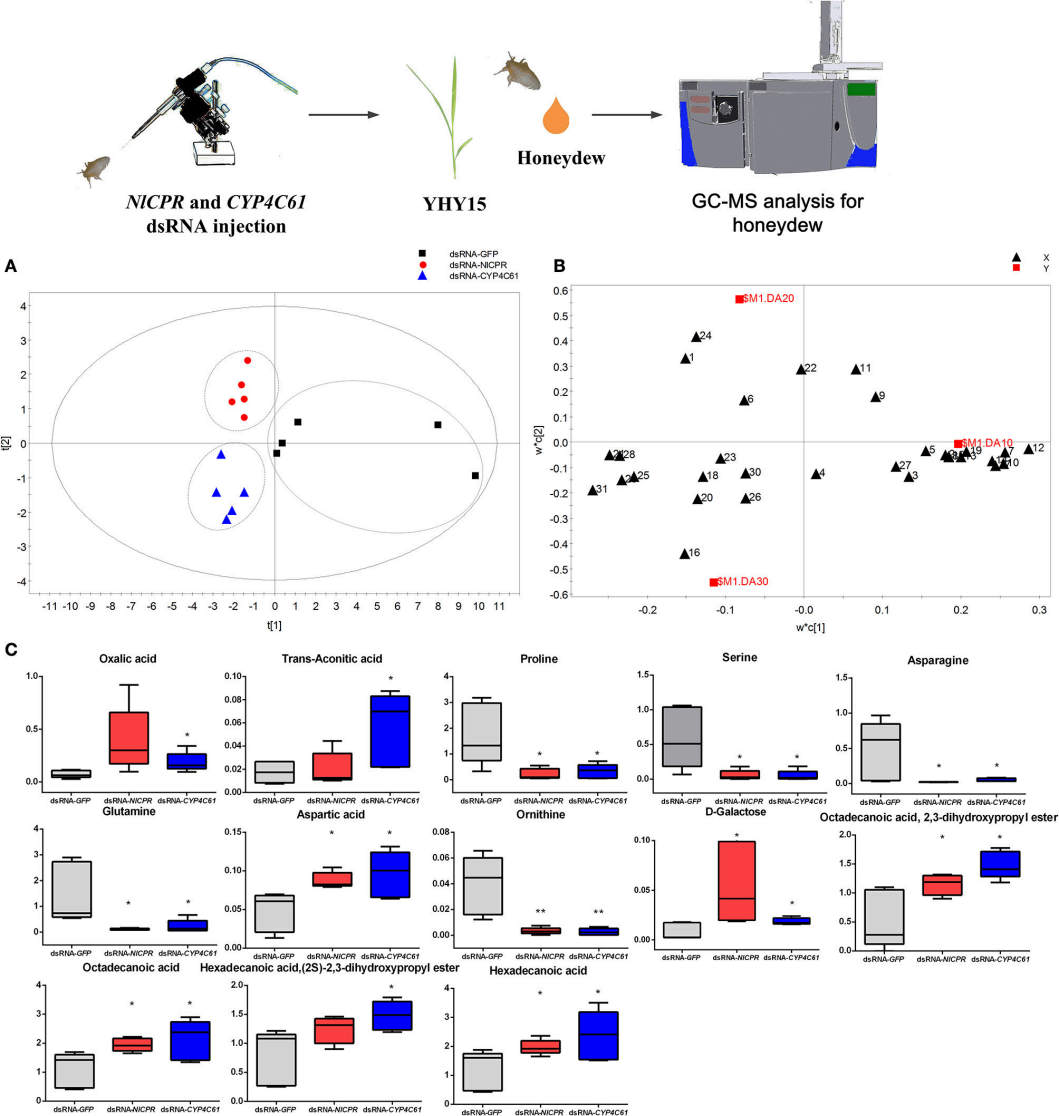
Metabolomics analysis of *NlCPR* and *CYP4C61* dsRNA-treated BPH honeydew on YHY15 plants. **(A)** PLS-DA score plots of dsRNA-treated BPH honeydew metabolites. **(B)** Loading plot of dsRNA-treated BPH honeydew metabolites. 1, Oxalic acid; 2, Valine; 3, Glycerol; 4, Phosphate; 5, Glycine; 6, Succinic acid; 7, Serine; 8, Threonine; 9, Malic acid; 10, Proline; 11, α-Hydroxypyruvic acid; 12, Ornithine; 13, Phenylalanine; 14, Asparagine; 15, α-Aminoadipic acid; 16, Trans-aconitic acid; 17, Glutamine; 18, D-Ribofuranose; 19, Shikimic acid; 20, 1,2,3-Propanetricarboxylic acid; 21, Aspartic acid; 22, D-Fructose; 23, D-Glucose; 24, D-Galactose; 25, Hexadecanoic acid; 26, Myo-Inositol; 27, Tryptophan; 28, Octadecanoic acid; 29, Hexadecanoic acid,(2S)-2,3-dihydroxypropyl ester; 30, α-D-Glucopyranoside; 31, Octadecanoic acid, 2,3-dihydroxypropyl ester. **(C)** Box plot of relative peak area data for honeydew metabolites. Each box plot shows the data distribution for each compound for all treatment groups. ^*^, ^**^ on the bars indicate significance at *P* < 0.05 and *P* < 0.01 (*t*-test), respectively, when compared with the control.

Three dsRNA-treated (ds*GFP*, ds*NlCPR*, and ds*CYP4C61*) honeydew metabolites were investigated by GC**–**MS. Figure S3B shows typical total ion chromatograms (TIC) of the honeydew. We identified a total of thirty-one metabolites (Table S2) by searching for matches between the mass spectra and standards in the MS library (NIST), and we compared retention times that matched with those of standard substances. Most of these identified compounds are primary metabolites, such as sugars, organic acids, and amino acids. We performed PLS-DA to investigate the various metabolites in honeydew among the three dsRNA treatments, and the results showed that the three dsRNA treatments could be clearly distinguished [R2X (cum) = 0.568, R2Y (cum) = 0.685, Q2 (cum) = 0.346]. The control (ds*GFP*) was mainly separated from the other dsRNA treatments in the PLS1 dimension (Figure 8A), and ds*NlCPR* was separated from ds*CYP4C61* in the PLS2 dimension. The PLS-DA loadings plot (Figure 8B) showed the variable influence on the separation. The significantly altered metabolites between the ds*GFP-*treated and ds*CPR-*treated or ds*CYP4C61*-treated insects are shown in Figure 8C. Overall, the levels of many amino acids were decreased when *NlCPR* or *CYP4C61* was knocked down, whereas oxalic acid, fatty acid, and fatty acid derivative levels were increased.

## Discussion

Previous studies have shown that rice leaf sheath extracts are toxic to BPHs and that resistant rice is more highly toxic than susceptible rice (Saxena and Okech, 1985; Stevenson et al., 1996). When sucrose solutions mixed with steam distillate extracts or ethanolic extracts of susceptible and resistant rice varieties were used to feed BPHs, the resistant rice extract was more toxic than that of the susceptible rice (Saxena and Okech, 1985; Stevenson et al., 1996). Additionally, the contents of identified compounds toxic to BPHs were found to be higher in the resistant rice than in the susceptible rice. For example, the levels of apigenin-C-glycoside, schaftoside, isoschaftoside, 3-nitraphthalic acid, β-sitosterol, stigmasterol, and campesterol were all higher in resistant than in susceptible rice, and feeding BPHs with these compounds decreased the their survival rate (Shigematsu et al., 1982; Stevenson et al., 1996; Zhang et al., 1999; Senthil-Nathan et al., 2007).

In the current study, we compared the survival rates of BPH biotypes 1 and Y when they were fed an artificial diet containing a leaf sheath extract of YHY15 rice and found a higher survival rate for biotype Y, which indicated that this biotype has an enhanced detoxification system. Metabolic resistance is an important strategy for insects to respond to chemical pressure (e.g., plant allelochemicals and pesticides). Indeed, under chemical toxin pressure, the ability to biotransform plant toxins is a major weapon that insects have evolved during their coevolutionary arms race with plants. Such an evolving detoxification system allows insects to adapt to plant toxin compounds and survive while feeding on host plants. Biotype Y evolved from biotype 1 and can survive on host plant YHY15; thus, we deduce that biotype Y evolved an enhanced ability to detoxify toxic compounds to adapt to resistant rice variety.

Glutathione S-transferase (GST), P450, and carboxylesterase (CE), which detoxify insecticides and some plant allelochemicals, have been recruited to increase insect resistance (Vogel et al., 2014). Insects have clearly adapted to the presence of plant toxins in their diets. Among several enzyme groups capable of inactivating plant toxins, P450s are key mediators of the hydroxylation and epoxidation required for efficient destruction and elimination of toxins prior to their adsorption (Schuler, 2011). The catalytic cycle of the P450 enzymes requires an electron donor, the CPR (Paine et al., 2005). CPR plays a key role in the P450 system by providing an electron to the catalytic cycle of the P450 enzymes. CPR can affect the P450 system by metabolizing toxic compounds. Previous studies have indicated that silencing CPR in oriental fruit flies, BPHs, carmine spider mites, and small BPHs may result in decreased activity of P450s, thus increasing susceptibility of these insects to insecticides (Huang et al., 2015; Liu et al., 2015; Shi et al., 2015; Zhang et al., 2016). Accordingly, downregulated CPR expression might result in a decreased capacity for electron transfer, which is necessary for the oxidizing ability of P450s.

To study the role of BPH biotype Y P450s in adapting to YHY15, we used RNAi to knockdown *NlCPR* by injecting *NlCPR* dsRNA into these BPHs and compared feeding behavior with that of the control BPHs (i.e., injected with *GFP* dsRNA). The results showed that when the *NlCPR* gene of biotype Y BPH was knocked down, honeydew excretion and weight gain were significantly lower compared to those of the control, which indicated inhibition of *NlCPR* knockdown BPHs feeding on YHY15. The EPG technique (Tjallingii, 1978) has been extensively used to catalog stylet activity in detail during insect feeding on host plants (Hao et al., 2008; Mutti et al., 2008; Seo et al., 2009). Using EPG, we tracked the stylet penetration behaviors of *NlCPR* dsRNA-treated BPHs at different time points. Waveforms showed that *NlCPR* knockdown BPH feedings was inhibited, reaching the most significant difference at 24 h. Based on these results, we concluded that decreasing P450 system activity affected the ability of biotype Y BPHs to adapt to YHY15. The P450 system may play a key role in metabolizing toxic compounds synthesized during the defense response of YHY15.

Many insect P450s are upregulated by both host plant toxins and pesticides in a tissue-specific manner, including in *Leptinotarsa decemlineata* (Zhu et al., 2016), *Spodoptera litura* (Wang R. et al., 2015a,b), *Spodoptera frugiperda* (Giraudo et al., 2015), *Bemisia tabaci* (Halon et al., 2015), and *H. zea* (Li et al., 2002). Previous studies have indicated that BPH P450 gene transcript levels are also induced by resistant rice varieties (Yang et al., 2005, 2007; Li et al., 2011). To investigate how BPH P450s respond to defense compounds of YHY15, we analyzed expression changes among 21 selected BPH genes from the P450 family and identified genes that were upregulated when BPHs fed on YHY15 and found that expression level of *CYP4C61* was upregulated when BPHs fed on YHY15. Silencing *CYP4C61* in BPHs caused reduced honeydew excretion and weight gain compared to the control. Moreover, EPG detection results showed that the feeding time of *CYP4C61* dsRNA-treated BPHs decreased from 12 to 72 h. According to these results, we deduce that CYP4C61 is involved in the metabolism of YHY15 plant allelochemicals and that knocking down *CYP4C61* inhibits BPH feeding and affects the ability of biotype Y to adapt to YHY15. Most P450s are generally considered to be expressed in the midgut and fat bodies, the sites of primary detoxification (Hodgson, 1985; Scott et al., 1998). qRT-PCR and sqPCR analyses showed that *CYP4C61* was mainly expressed in the fat body and midgut; therefore, it might play a role in detoxification.

The metabolic changes induced by these genes need to be examined via metabolomics (Nicholson and Lindon, 2008). As qualitative and quantitative analyses of honeydew can offer valuable information about sucking sites and rates (Sogawa, 1982), changes in honeydew metabolites when BPHs fed on different rice varieties can dynamically reflect the utilization of rice phloem sap in the BPH digestive tract as well as BPH metabolic physiology. Previous studies have shown that compared to TN1, BPHs feeding on YHY15 produce honeydew with a lower amino acid content due to enhanced amino acid absorption (Peng et al., 2016). In the present study, the levels of many amino acids in the *NlCPR* and *CYP4C61* dsRNA-treated samples were lower than those in the control samples, indicating that feeding was inhibited and amino acid absorption enhanced. The contents of fatty acids and fatty acid derivatives in *NlCPR*-knockdown and *CYP4C61*-knockdown BPHs were increased compared to those of the control.

In BPHs, CYP4C61 may participate in allelochemical detoxification. However, variations in the detoxification capability may exist between biotypes 1 and Y. In the P450 enzyme family, a single amino acid change may lead to two closely related enzymes acting on the same substrate, causing regioselectivity of the hydroxylation change (Schalk and Croteau, 2000; Sezutsu et al., 2013). Duplication of P450 genes is also considered to contribute to metabolic resistance or host adaptation (Wen et al., 2006; Emerson et al., 2008). Biotype Y CYP4C61 differs by one amino acid in predicted SRS regions. In this study, the transcript level of biotype Y *CYP4C61* induced by YHY15 was higher than that of biotype 1. These results show that changes in amino acids and expression may also result in changes in metabolism. Further investigation needs to be performed to uncover the mechanism underlying this phenomenon.

**Figure 9:**
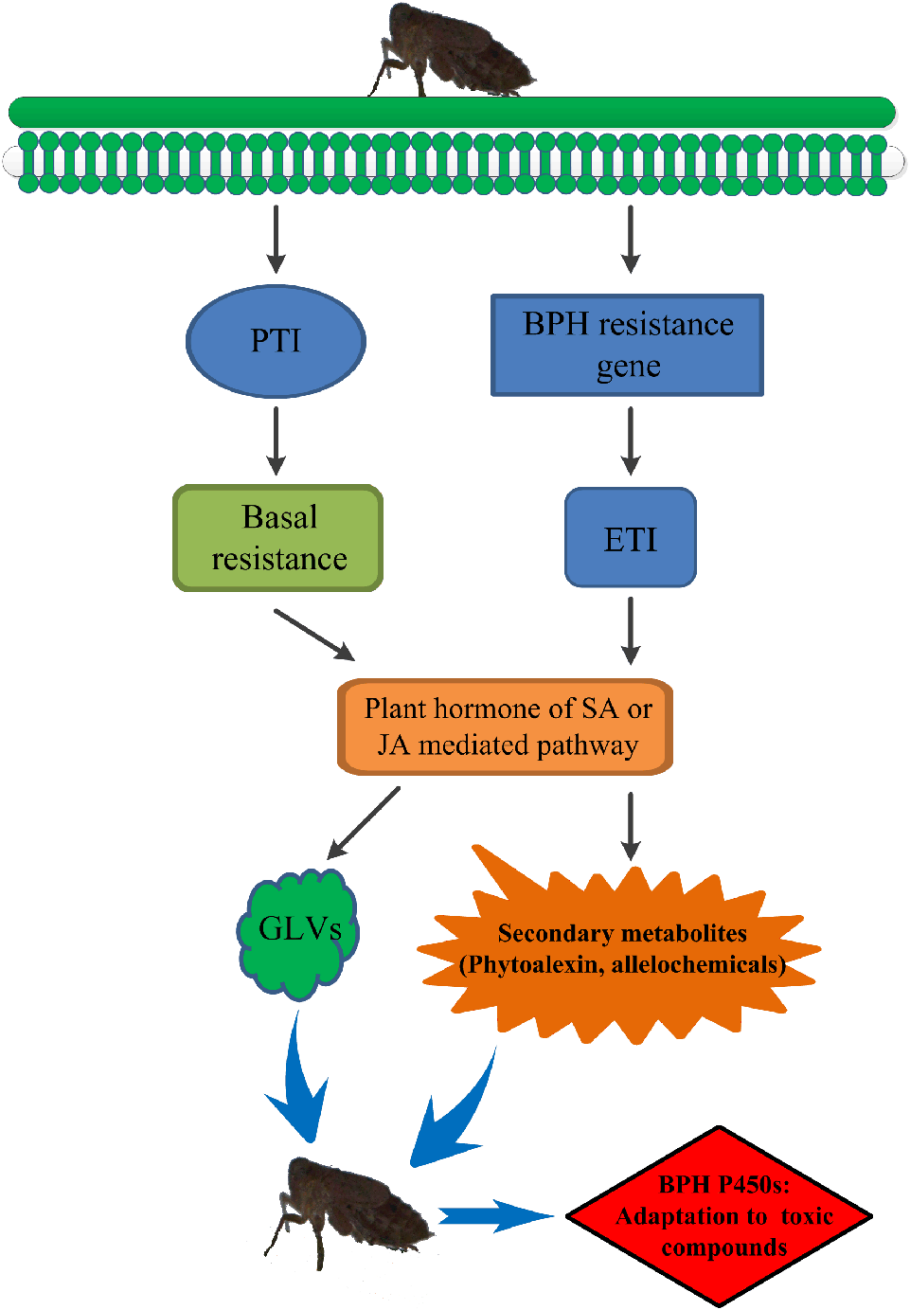
The preliminary model of rice resistance via allelochemicals to drive evolution of BPH P450 enzymes. PTI, PRR (pattern recognition receptor)-triggered immunity; ETI, effector-triggered immunity; GLVs, green leaf volatiles. BPH feeding induces PRR-triggered immunity (PTI) and effector-triggered immunity (ETI). PTI is the basal immune status that is effective against a broad spectrum of pathogens or herbivores and induces the SA or JA signaling pathway. ETI triggers a series of BPH-mediated immune responses and profoundly promotes basal resistance, including inducing the SA or JA signaling pathway. Plant hormones such as SA or JA mediate phytoalexins or secondary metabolites to resist BPH infestation. BPH detoxifies allelochemicals to adapt to its host plant using its detoxification system consisting of the P450 family of enzymes.

Secondary metabolites are derived from pathways that are transcriptionally induced by the PAMPs (pathogen- or microbe-associated molecular patterns) receptor-activated shikimate pathway (Cheng et al., 2013; Grant et al., 2013). Previous metabolomic studies have indicated activation of the shikimate pathway in the resistant rice varieties B5 (containing *BPH14* and *BPH15*) and YHY15, thus producing secondary metabolites to combat against BPH infestation (Liu et al., 2010; Peng et al., 2016). Resistance gene-regulated synthesis of secondary metabolites may be achieved through plant hormones. For example, salicylic acid (SA) is proven to be involved in *BPH14-, BPH29-*, and *BPH9-*mediated defense response (Du et al., 2009; Wang Y. et al., 2015; Zhao et al., 2016), and jasmonic acid (JA) is associated with *BPH9* (Zhao et al., 2016). SA has also been found to be required for accumulation of phytoalexins, camalexins (Zhao and Last, 1996; Zhou et al., 1998; Ferrari et al., 2003), diterpenoid phytoalexins (DPs) (Akagi et al., 2014), and isoflavonoids (Durango et al., 2013), and JA acts as a conserved elicitor of plant secondary metabolism and responds to insect attacks and abiotic and biotic stresses (De Geyter et al., 2012; Song et al., 2014). Increased levels of JA and phytoalexins were found in rice infested with white-backed planthoppers or *Fusarium fujikuroi* and *Magnaporthe oryzae* (Kanno et al., 2012; Duan et al., 2014; Siciliano et al., 2015). BPH resistance genes might also mediate phytoalexin or secondary metabolite production by regulating plant hormones such as SA or JA to resist BPH infestation. In our study, exposure to toxic allelochemicals due to extended periods of feeding on resistant rice varieties resulted in evolution of the BPH detoxification system (Figure 9).

In our current research, we demonstrate that P450 enzymes participate in BPH adaptation to resistant rice strain YHY15. Among the enzymes assessed, we show that CYP4C61 plays a key role in this adaptation to YHY15. Overall, our findings lead to a better understanding of the molecular mechanism underlying the interaction between resistant rice and BPHs.

## Author contributions

GH and LP: conceived and designed the work; LP: performed the experiments and wrote the paper; YZ: helped to perform the GC–MS experiment and revised the manuscript; HW performed the tissue preparation experiment and contributed materials of plants and insects; CS helped to perform the leaf sheath ethanol extract experiment; XS and YM: contributed materials of plants and insects. All authors gave the final approval for publication.

### Conflict of interest statement

The authors declare that the research was conducted in the absence of any commercial or financial relationships that could be construed as a potential conflict of interest.

## References

[B1] Akagi A. Fukushima S. Okada K. Jiang C. J. Yoshida R. Nakayama A.. (2014). WRKY45-dependent priming of diterpenoid phytoalexin biosynthesis in rice and the role of cytokinin in triggering the reaction. Plant Mol. Biol. 86, 171–183. 10.1007/s11103-014-0221-x 25033935 PMC4133022

[B2] Alamgir K. M. Hojo Y. Christeller J. T. Fukumoto K. Isshiki R. Shinya T.. (2016). Systematic analysis of rice (*Oryza sativa*) metabolic responses to herbivory. Plant Cell Environ. 39, 453–466. 10.1111/pce.12640 26386366

[B3] Bass C. Carvalho R. A. Oliphant L. Puinean A. M. Field L. M. Nauen R.. (2011). Overexpression of a cytochrome P450 monooxygenase, CYP6ER1, is associated with resistance to imidacloprid in the brown planthopper, *Nilaparvata lugens*. Insect Mol. Biol. 20, 763–773. 10.1111/j.1365-2583.2011.01105.x 21929695

[B4] Bass C. Zimmer C. T. Riveron J. M. Wilding C. S. Wondji C. S. Kaussmann M.. (2013). Gene amplification and microsatellite polymorphism underlie a recent insect host shift. Proc. Natl. Acad. Sci. U S A. 110, 19460–19465. 10.1073/pnas.1314122110 24218582 PMC3845143

[B5] Chen W. Gao Y. Xie W. Gong L. Lu K. Wang W.. (2014). Genome-wide association analyses provide genetic and biochemical insights into natural variation in rice metabolism. Nat. Genet. 46, 714–721. 10.1038/ng.3007 24908251

[B6] Cheng X. Zhu L. He G. (2013). Towards understanding of molecular interactions between rice and the brown planthopper. Mol. Plant 6, 621–634. 10.1093/mp/sst030 23396040

[B7] Claridge M. F. Hollander J. D. (1980). The “biotypes” of the rice brown planthopper, *Nilaparvatalugens*. Entomol. Exp. Appl. 27, 23–30. 10.1111/j.1570-7458.1980.tb02942.x19795662

[B8] De Geyter N. Gholami A. Goormachtig S. Goossens A. (2012). Transcriptional machineries in jasmonate-elicited plant secondary metabolism. Trends Plant Sci. 17, 349–359. 10.1016/j.tplants.2012.03.001 22459758

[B9] Du B. Zhang W. Liu B. Hu J. Wei Z. Shi Z.. (2009). Identification and characterization of Bph14, a gene conferring resistance to brown planthopper in rice. Proc. Natl. Acad. Sci. U.S.A. 106, 22163–22168. 10.1073/pnas.0912139106 20018701 PMC2793316

[B10] Duan L. Liu H. Li X. Xiao J. Wang S. (2014). Multiple phytohormones and phytoalexins are involved in disease resistance to *Magnaporthe oryzae* invaded from roots in rice. Physiol Plant. 152, 486–500. 10.1111/ppl.12192 24684436

[B11] Durango D. Pulgarin N. Echeverri F. Escobar G. Quiñones W. (2013). Effect of salicylic acid and structurally related compounds in the accumulation of phytoalexins in cotyledons of common bean (*Phaseolus vulgaris* L.) cultivars. Molecules 18, 10609–10628. 10.3390/molecules180910609 24002137 PMC6269966

[B12] Emerson J. J. Cardoso-Moreira M. Borevitz J. O. Long M. (2008). Natural selection shapes genome-wide patterns of copy-number polymorphism in Drosophila melanogaster. Science 320, 1629–1631. 10.1126/science.1158078 18535209

[B13] Ferrari S. Plotnikova J. M. De Lorenzo G. Ausubel F. M. (2003). Arabidopsis local resistance to *Botrytis cinerea* involves salicylic acid and camalexin and requires EDS4 and PAD2, but not SID2, EDS5 or PAD4. Plant J. 35, 193–205. 10.1046/j.1365-313X.2003.01794.x 12848825

[B14] Giraudo M. Hilliou F. Fricaux T. Audant P. Feyereisen R. Le Goff G. (2015). Cytochrome P450s from the fall armyworm (*Spodoptera frugiperda*): responses to plant allelochemicals and pesticides. Insect Mol. Biol. 24, 115–128. 10.1111/imb.12140 25315858

[B15] Grant M. R. Kazan K. Manners J. M. (2013). Exploiting pathogens' tricks of the trade for engineering of plant disease resistance: challenges and opportunities. Microb. Biotechnol. 6, 212–222. 10.1111/1751-7915.12017 23279915 PMC3815916

[B16] Halon E. Eakteiman G. Moshitzky P. Elbaz M. Alon M. Pavlidi N.. (2015). Only a minority of broad-range detoxification genes respond to a variety of phytotoxins in generalist *Bemisia tabaci* species. Sci Rep. 5:17975. 10.1038/srep17975 26655836 PMC4674796

[B17] Hao P. Liu C. Wang Y. Chen R. Tang M. Du B.. (2008). Herbivore-induced callose deposition on the sieve plates of rice: an important mechanism for host resistance. Plant Physiol. 146, 1810–1820. 10.1104/pp.107.111484 18245456 PMC2287352

[B18] Heidel-Fischer H. M. Vogel H. (2015). Molecular mechanisms of insect adaptation to plant secondary compounds. Curr. Opin. Insect Sci. 8, 8–14. 10.1016/j.cois.2015.02.004 32846688

[B19] Hodgson E. (1985). Microsomal mono-oxygenases, in Comprehensive Insect Physiology Biochemistry and Pharmacology, eds Kerkut G. A. Gilbert L. I. (Oxford: Pergamon Press), 225–321.

[B20] Horgan F. (2009). Mechanisms of resistance: a major gap in understanding planthopper-rice interactions, in Planthoppers: New Threats to the Sustainability of Intensive Rice Production Systems in Asia, eds Heong K. L. Hardy B. B. (Los Baños: International Rice Research Institute), 281–302.

[B21] Huang Y. Lu X. P. Wang L. L. Wei D. Feng Z. J. Zhang Q.. (2015). Functional characterization of NADPH-cytochrome P450 reductase from Bactrocera dorsalis: Possible involvement in susceptibility to malathion. Sci Rep. 5:18394. 10.1038/srep18394 26681597 PMC4683403

[B22] Jena K. K. Kim S. M. (2010). Current status of brown planthopper (BPH) resistance and genetics. Rice 3, 161–171. 10.1007/s12284-010-9050-y

[B23] Jing S. Liu B. Peng L. Peng X. Zhu L. Fu Q.. (2012). Development and use of EST-SSR markers for assessing genetic diversity in the brown planthopper (*Nilaparvata lugens* Stål). Bull. Entomol. Res. 102, 113–122. 10.1017/S0007485311000435 21896240

[B24] Jing S. Zhao Y. Du B. Chen R. Zhu L. He G. (2017). Genomics of interaction between the brown planthopper and rice. Curr. Opin. Insect Sci. 19, 82–87. 10.1016/j.cois.2017.03.005 28521948

[B25] Kanno H. Hasegawa M. Kodama O. (2012). Accumulation of salicylic acid, jasmonic acid and phytoalexins in rice, *Oryza sativa*, infested by the white-backed planthopper, *Sogatella furcifera* (Hemiptera: Delphacidae). Appl. Entomol. Zool. 47, 27–34. 10.1007/s13355-011-0085-3

[B26] Kiritani K. (1979). Pest management in rice. Annu Rev Entomol. 24, 279–312. 10.1146/annurev.en.24.010179.001431

[B27] Lao S. H. Huang X. H. Huang H. J. Liu C. W. Zhang C. X. Bao Y. Y. (2015). Genomic and transcriptomic insights into the cytochrome P450 monooxygenase gene repertoire in the rice pest brown planthopper, *Nilaparvata lugens*. Genomics 106, 301–309. 10.1016/j.ygeno.2015.07.010 26234643

[B28] Li J. Chen Q. Wang L. Liu J. Shang K. Hua H. (2011). Biological effects of rice harbouring Bph14 and Bph15 on brown planthopper, *Nilaparvata lugens*. Pest Manag. Sci. 67, 528–534. 10.1002/ps.2089 21254325

[B29] Li X. Baudry J. Berenbaum M. R. Schuler M. A. (2004). Structural and functional divergence of insect CYP6B proteins: from specialist to generalist cytochrome P450. Proc. Natl. Acad. Sci. U.S.A. 101, 2939–2944. 10.1073/pnas.0308691101 14981232 PMC365724

[B30] Li X. Berenbaum M. R. Schuler M. A. (2002). Plant allelochemicals differentially regulate *Helicoverpa zea* cytochrome P450 genes. Insect Mol. Biol. 343–351. 10.1046/j.1365-2583.2002.00341.x 12144700

[B31] Liu C. Hao F. Hu J. Zhang W. Wan L. Zhu L.. (2010). Revealing different systems responses to brown planthopper infestation for pest susceptible and resistant rice plants with the combined metabonomic and gene-expression analysis. J. Proteome Res. 9, 6774–6785. 10.1021/pr100970q 20936879

[B32] Liu S. Liang Q. M. Zhou W. W. Jiang Y. D. Zhu Q. Z. Yu H.. (2015). RNA interference of NADPH–cytochrome P450 reductase of the rice brown planthopper, *Nilaparvata lugens*, increases susceptibility to insecticides. Pest Manag. Sci. 71, 32–39. 10.1002/ps.3760 24515640

[B33] Lv W. Du B. Shangguan X. Zhao Y. Pan Y. Zhu L.. (2014). BAC and RNA sequencing reveal the brown planthopper resistance gene BPH15 in a recombination cold spot that mediates a unique defense mechanism. BMC Genomics 15:674. 10.1186/1471-2164-15-674 25109872 PMC4148935

[B34] Mao W. Schuler M. A. Berenbaum M. R. (2007). Cytochrome P450s in Papilio multicaudatus and the transition from oligophagy to polyphagy in the Papilionidae. Insect Mol. Biol. 16, 481–490. 10.1111/j.1365-2583.2007.00741.x 17651237

[B35] Mao W. Schuler M. A. Berenbaum M. R. (2011). CYP9Q-mediated detoxification of acaricides in the honey bee (*Apis mellifera*). Proc. Natl. Acad. Sci. U.S.A. 108, 12657–12662. 10.1073/pnas.1109535108 21775671 PMC3150950

[B36] Mutti N. S. Louis J. Pappan L. K. Pappan K. Begum K. Chen M. S.. (2008). A protein from the salivary glands of the pea aphid, *Acyrthosiphon pisum*, is essential in feeding on a host plant. Proc. Natl. Acad. Sci. U.S.A. 105, 9965–9969. 10.1073/pnas.0708958105 18621720 PMC2481341

[B37] Nicholson J. K. Lindon J. C. (2008). Systems biology: metabonomics. Nature 455, 1054–1056. 10.1038/4551054a 18948945

[B38] Paine M. J. Scrutton N. S. Munro A. W. Gutierrez A. Roberts G. C. Wolf C. R. (2005). Electron transfer partners of cytochrome P450, in Cytochrome P450: Structure, Mechanism, and Biochemistry ed Ortiz De Montellano P. (New York, NY: Kluwer Academic/Plenum Publishers), 115–138.

[B39] Peng L. Zhao Y. Wang H. Zhang J. Song C. Shangguan X. (2016). Comparative metabolomics of the interaction between rice and the brown planthopper. Metabolomics 12, 132 10.1007/s11306-016-1077-7

[B40] Rani P. U. Jyothsna Y. (2010). Biochemical and enzymatic changes in rice plants as a mechanism of defense. Acta Physiol. Plant. 32, 695–701. 10.1007/s11738-009-0449-2

[B41] Raucy J. L. Allen S. W. (2001). Recent advances in P450 research. Pharmacogenomics J. 1, 178–186. 10.1038/sj.tpj.6500044 11908754

[B42] Rewitz K. F. Styrishave B. Løbner-Olesen A. Andersen O. (2006). Marine invertebrate cytochrome P450: emerging insights from vertebrate and insect analogies. Comp. Biochem. Physiol. C Toxicol. Pharmacol. 143, 363–381. 10.1016/j.cbpc.2006.04.001 16769251

[B43] Sandstrom P. Welch W. H. Blomquist G. J. Tittiger C. (2006). Functional expression of a bark beetle cytochrome P450 that hydroxylates myrcene to ipsdienol. Insect Biochem. Mol. Biol. 36, 835–845. 10.1016/j.ibmb.2006.08.004 17046597

[B44] Saxena R. C. Okech S. H. (1985). Role of plant volatiles in resistance of selected rice varieties to brown planthopper, *Nilaparvata lugens* (Stål) (Homoptera: Delphacidae). J. Chem. Ecol. 11, 1601–1616. 10.1007/BF01012115 24311329

[B45] Schalk M. Croteau R. (2000). A single amino acid substitution (F363I) converts the regiochemistry of the spearmint (–)-limonene hydroxylase from a C6-to a C3-hydroxylase. Proc. Natl. Acad. Sci. U.S.A. 97, 11948–11953. 10.1073/pnas.97.22.11948 11050228 PMC17275

[B46] Schuler M. A. (2011). P450s in plant–insect interactions. Biochim. Biophys. Acta 1814, 36–45. 10.1016/j.bbapap.2010.09.012 20883828

[B47] Scott J. G. Liu N. Wen Z. (1998). Insect cytochromes P450: diversity, insecticide resistance and tolerance to plant toxins. Comp. Biochem. Physiol. 121, 147–155. 10.1016/S0742-8413(98)10035-X 9972456

[B48] Senthil-Nathan S. (2013). Physiological and biochemical effect of neem and other Meliaceae plants secondary metabolites against Lepidopteran insects. Front Physiol. 4:359. 10.3389/fphys.2013.00359 24391591 PMC3868951

[B49] Senthil-Nathan S. Choi M. Y. Paik C. H. Seo H. Y. Kalaivani K. (2009a). Toxicity and physiological effects of neem pesticides applied to rice on the *Nilaparvata lugens* Stål, the brown planthopper. Ecotoxicol. Environ. Saf. 72, 1707–1713. 10.1016/j.ecoenv.2009.04.024 19500844

[B50] Senthil-Nathan S. Choi M. Y. Paik C. H. Seo H. Y. Kim J. D. Kang S. M. (2007). The toxic effects of neem extract and azadirachtin on the brown planthopper, *Nilaparvata lugens* (Stål) (BPH) (Homoptera: Delphacidae). Chemosphere 67, 80–88. 10.1016/j.chemosphere.2006.09.045 17113126

[B51] Senthil-Nathan S. Choi M. Y. Seo H. Y. Paik C. H. Kalaivani K. Kim J. D. (2008). Effect of azadirachtin on acetylcholinesterase (AChE) activity and histology of the brown planthopper *Nilaparvata lugens* (Stål). Ecotox Environ. Saf. 70, 244–250. 10.1016/j.ecoenv.2007.07.005 17765967

[B52] Senthil-Nathan S. Kalaivani K. Choi M. Y. Paik C. H. (2009b). Effects of jasmonic acid-induced resistance in rice on the plant brownhopper, *Nilaparvata lugens* Stål (Homoptera: Delphacidae). Pestic. Biochem. Physiol. 95, 77–84. 10.1016/j.pestbp.2009.07.001

[B53] Seo B. Y. Kwon Y. H. Jung J. K. Kim G. H. (2009). Electrical penetration graphic waveforms in relation to the actual positions of the stylet tips of *Nilaparvata lugens* in rice tissue. J. Asia Pac. Entomol. 12, 89–95. 10.1016/j.aspen.2009.02.002

[B54] Sezutsu H. Le Goff G. Feyereisen R. (2013). Origins of P450 diversity. Philos. Trans. R. Soc. B 368:20120428. 10.1098/rstb.2012.0428 23297351 PMC3538418

[B55] Shi L. Zhang J. Shen G. Xu Z. Wei P. Zhang Y.. (2015). Silencing NADPH-cytochrome P450 reductase results in reduced acaricide resistance in *Tetranychus cinnabarinus* (Boisduval). Sci. Rep. 5:15581. 10.1038/srep15581 26493678 PMC4616063

[B56] Shigematsu Y. Murofushi N. Ito K. Kaneda C. Kawabe S. Takahashi N. (1982). Sterols and asparagine in the rice plant, endogenous factors related to resistance against the brown planthopper (*Nilaparvata lugens*). Agric. Biol. Chem. 46, 2877–2879.

[B57] Siciliano I. Amaral Carneiro G. Spadaro D. Garibaldi A. Gullino M. L. (2015). Jasmonic acid, abscisic acid, and salicylic acid are involved in the phytoalexin responses of rice to Fusarium fujikuroi, a high gibberellin producer pathogen. J. Agric. Food Chem. 63, 8134–8142. 10.1021/acs.jafc.5b03018 26323788

[B58] Sogawa K. (1982). The rice brown planthopper: feeding physiology and host plant interactions. Annu. Rev. Entomol. 27, 49–73. 10.1146/annurev.en.27.010182.000405

[B59] Sõgawa K. Pathak M. D. (1970). Mechanisms of brown planthopper resistance in Mudgo variety of rice (Hemiptera: Delphacidae). Appl. Entomol. Zool. 5, 145–158. 10.1303/aez.5.145

[B60] Song S. Qi T. Wasternack C. Xie D. (2014). Jasmonate signaling and crosstalk with gibberellin and ethylene. Curr. Opin. Plant Biol. 21, 112–119. 10.1016/j.pbi.2014.07.005 25064075

[B61] Stevenson P. C. Kimmins F. M. Grayer R. J. Raveendranath S. (1996). Schaftosides from rice phloem as feeding inhibitors and resistance factors to brown planthoppers, *Nilaparvata lugens*. Entomol. Exp. Appl. 80, 246–249. 10.1111/j.1570-7458.1996.tb00928.x

[B62] Tjallingii W. F. (1978). Electronic recording of penetration behaviour by aphids. Entomol. Exp. Appl. 24, 721–730. 10.1111/j.1570-7458.1978.tb02836.x

[B63] Vogel H. Musser R. O. Celorio-Mancera M. L. (2014). Transcriptome responses in herbivorous insects towards host plant and toxin feeding, in Annual Plant Reviews; Plant Insect Interactions, eds Voelckel C. Jander G. (Chichester: John Wiley & Sons, Ltd.), 197–233.

[B64] Wang R. L. Li J. Staehelin C. Xin X. W. Su Y. J. Zeng R. S. (2015a). Expression analysis of two P450 monooxygenase genes of the tobacco cutworm moth (*Spodoptera litura*) at different developmental stages and in response to plant allelochemicals. J. Chem. Ecol. 41, 111–119. 10.1007/s10886-014-0540-z 25547988

[B65] Wang R. L. Staehelin C. Xia Q. Q. Su Y. J. Zeng R. S. (2015b). Identification and Characterization of CYP9A40 from the Tobacco Cutworm Moth (*Spodoptera litura*), a cytochrome P450 gene induced by plant allelochemicals and insecticides. Int. J. Mol. Sci. 16, 22606–22620. 10.3390/ijms160922606 26393579 PMC4613326

[B66] Wang Y. Cao L. Zhang Y. Cao C. Liu F. Huang F.. (2015). Map-based cloning and characterization of BPH29, a B3 domain-containing recessive gene conferring brown planthopper resistance in rice. J. Exp. Bot. 66, 6035–6045. 10.1093/jxb/erv318 26136269 PMC4566989

[B67] Wen Z. Rupasinghe S. Niu G. Berenbaum M. R. Schuler M. A. (2006). CYP6B1 and CYP6B3 of the black swallowtail (*Papilio polyxenes*): adaptive evolution through subfunctionalization. Mol. Biol. Evol. 23, 2434–2443. 10.1093/molbev/msl118 16984951

[B68] Wittstock U. Gershenzon J. (2002). Constitutive plant toxins and their role in defense against herbivores and pathogens. Curr. Opin. Plant Biol. 5, 300–307. 10.1016/S1369-5266(02)00264-9 12179963

[B69] Yang Z. Yang H. He G. (2007). Cloning and characterization of two cytochrome P450 CYP6AX1 and CYP6AY1 cDNAs from *Nilaparvata lugens* Stål (Homoptera: Delphacidae). Arch. Insect Biochem. Physiol. 64, 88–99. 10.1002/arch.20162 17212353

[B70] Yang H. You A. Yang Z. Zhang F. He R. Zhu L.. (2004). High-resolution genetic mapping at the Bph15 locus for brown planthopper resistance in rice (*Oryza sativa* L.). Theor. Appl. Genet. 110, 182–191. 10.1007/s00122-004-1844-0 15549231

[B71] Yang Z. Zhang F. He Q. He G. (2005). Molecular dynamics of detoxification and toxin-tolerance genes in brown planthopper (*Nilaparvata lugens Stål*., Homoptera: Delphacidae) feeding on resistant rice plants. Arch. Insect Biochem. Physiol. 59, 59–66. 10.1002/arch.20055 15898115

[B72] Yoshihara T. Sogawa K. Pathak M. D. Juliano B. O. Sakamura S. (1980). Oxalic acid as a sucking inhibitor of the brown planthopper in rice (Delphacidae, Homoptera). Entomol. Exp. Appl. 27, 149–155. 10.1111/j.1570-7458.1980.tb02959.x

[B73] Zhang G. Zhang W. Lian B. Gu L. Zhou Q. Liu T. X. (1999). Insecticidal effects of extracts from two rice varieties to brown planthopper, *Nilaparvata lugens*. J. Chem. Ecol. 25, 1843–1853. 10.1023/A:1020981716293

[B74] Zhang Y. Wang Y. Wang L. Yao J. Guo H. Fang J. (2016). Knockdown of NADPH-cytochrome P450 reductase results in reduced resistance to buprofezin in the small brown planthopper, *Laodelphax striatellus* (fallén). Pestic. Biochem. Physiol. 127, 21–27. 10.1016/j.pestbp.2015.08.006 26821654

[B75] Zhao J. Last R. L. (1996). Coordinate regulation of the tryptophan biosynthetic pathway and indolic phytoalexin accumulation in Arabidopsis. Plant Cell. 8, 2235–2244. 10.1105/tpc.8.12.2235 8989880 PMC161348

[B76] Zhao Y. Huang J. Wang Z. Jing S. Wang Y. Ouyang Y. (2016). Allelic diversity in an NLR gene BPH9 enables rice to combat planthopper variation. Proc. Natl Acad. Sci. U.S.A. 113, 12850–12855. 10.1073/pnas.1614862113 PMC5111712 27791169

[B77] Zhou N. Tootle T. L. Tsui F. Klessig D. F. Glazebrook J. (1998). PAD4 functions upstream from salicylic acid to control defense responses in Arabidopsis. Plant Cell. 10, 1021–1030. 10.1105/tpc.10.6.1021 9634589 PMC144042

[B78] Zhu F. Moural T. W. Nelson D. R. Palli S. R. (2016). A specialist herbivore pest adaptation to xenobiotics through up-regulation of multiple Cytochrome P450s. Sci Rep. 6:20421. 10.1038/srep20421 26861263 PMC4748221

