# Supplementary material for: Functional Study of Cytochrome P450 Enzymes from the Brown Planthopper (Nilaparvata lugens Stål) to Analyze Its Adaptation to BPH-Resistant Rice
Source: Front Physiol. 2017 Nov 30;8:972. doi: 10.3389/fphys.2017.00972 (PMC5714877; doi:10.3389/fphys.2017.00972)
Supplement: Supplementary file 1 [file Image1.PDF]

Supplementary material: Figures

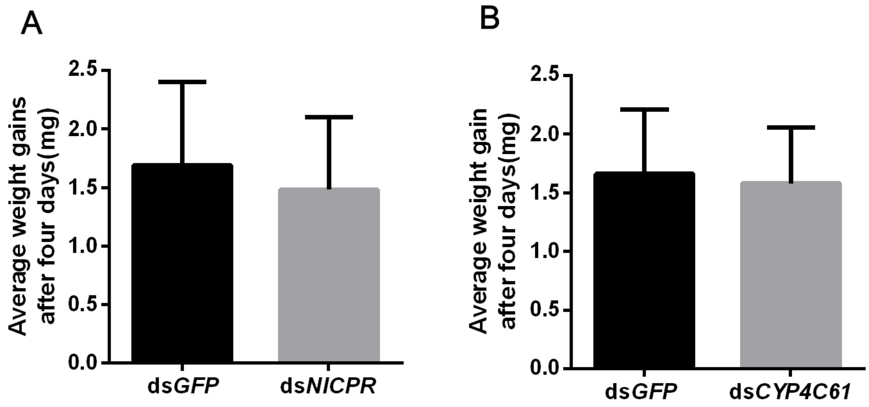

Figure S1. Weight gain of dsRNA-treated BPH feeding on TN1. (A) Weight gain of *NlCPR* dsRNA-treated BPH feeding on TN1. (B) Weight gain of *CYP4C61* dsRNA-treated BPH feeding on TN1.

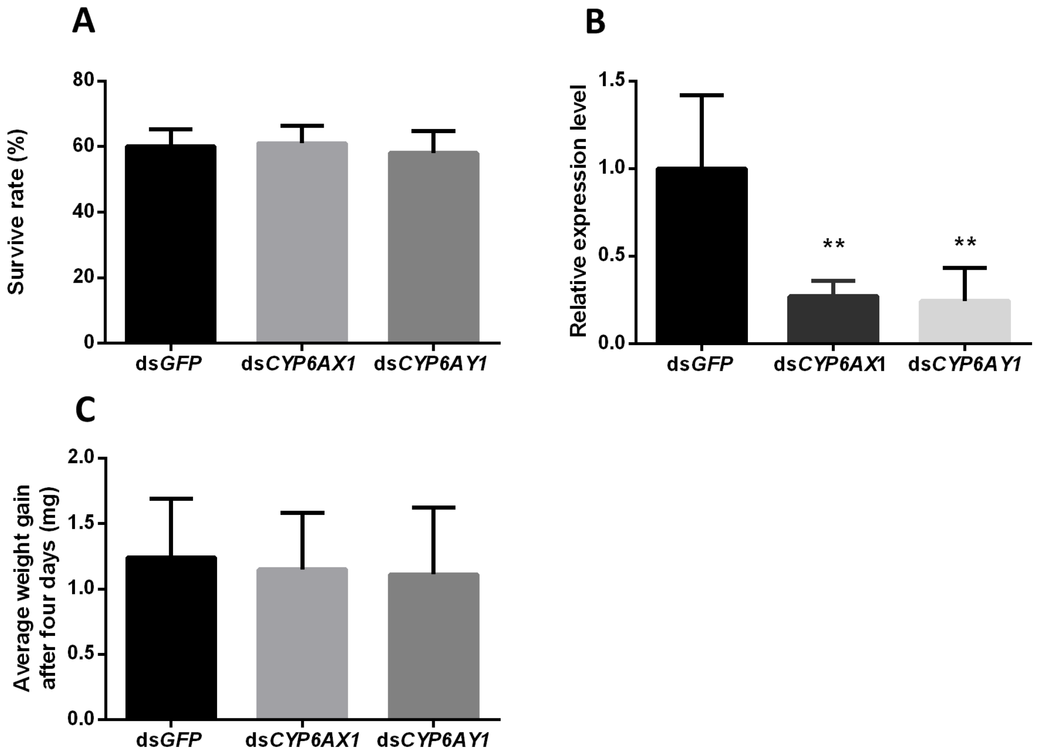

Figure S2. Analysis of the knockdown of biotype Y BPH *CYP6AX1* and biotype Y BPH *CYP6AY1*. (A) Survival rates of biotype Y BPH treated with *CYP6AX1* and *CYP6AY1* and *GFP* dsRNA. (B) qRT-PCR analysis of the effects of *CYP6AX1* and *CYP6AY1* knockdown. (C) Weight gain of dsRNA-treated BPH. ** on the bars indicates significance at $P < 0.01$(t-test) when compared with the control.

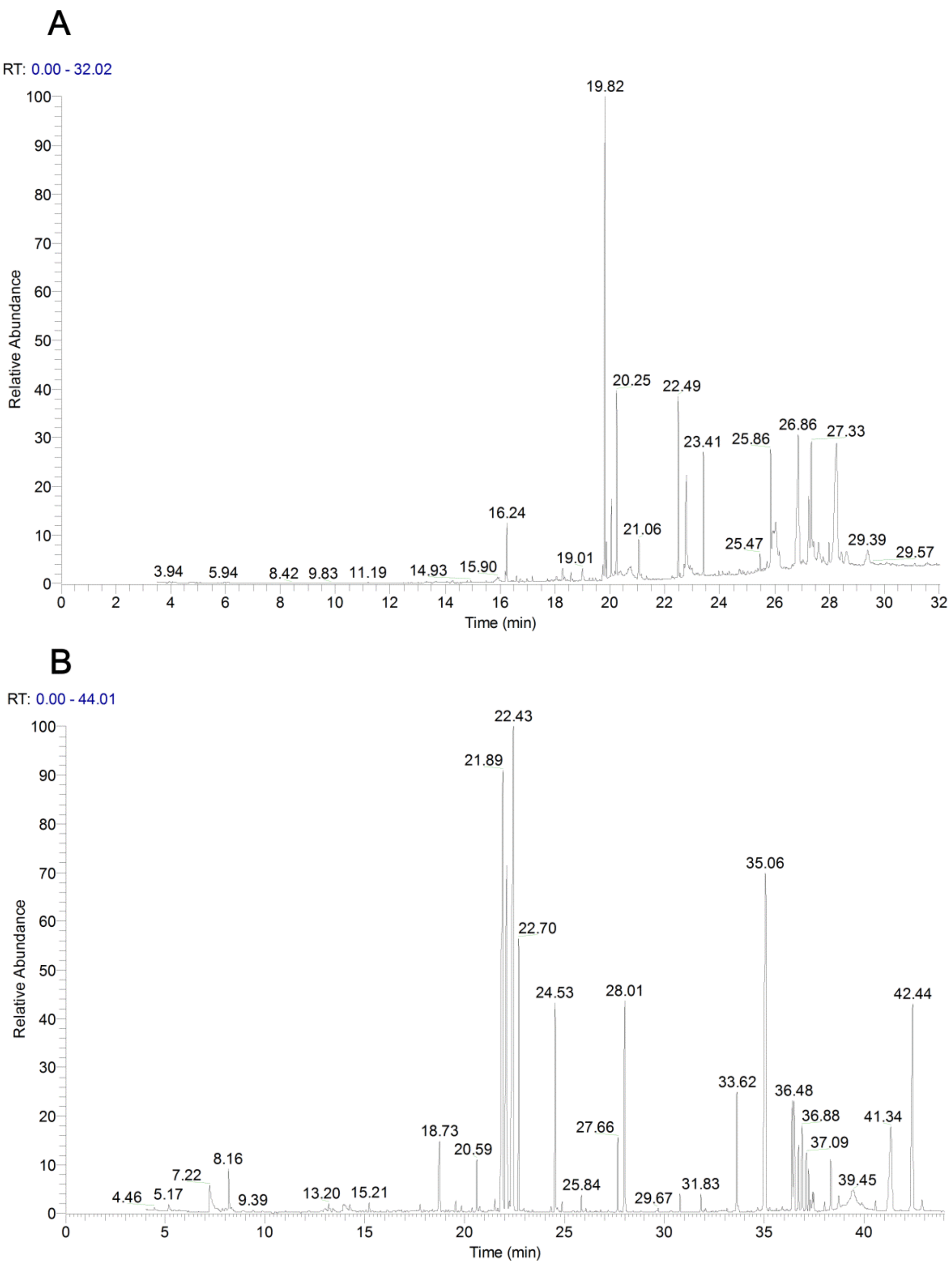

Figure S3. GC–MS total ion chromatograms (TIC) of the rice leaf sheath extract and honeydew samples. (A) TIC of the rice leaf sheath extract sample. (B) TIC of the honeydew sample.

```
BY CYP4C61  1    ATGAAGATGATTACTTTAATATTGACTTCGTTTGCTCTCGCTATCATTGTAACATACCTT
B1 CYP4C61  1    ............................................................
CYP4C61v1   1    ............................................................
CYP4C61v2   1    ~~~~~~...........C..........................................

BY CYP4C61  61   GTTAAGTTGTCCTATAGAAGATATCAATTTCTGAGAAAGCTCAAGGATTGCCTGGGCCT
B1 CYP4C61  61   ..A...C............................G........................
CYP4C61v1   61   ..A...C............................G........................
CYP4C61v2   55   ......C.....................................................

BY CYP4C61  121  AAGGCTTATCCAATTGTTGGAGACTCATTGGAAATGCTATATTTGAAGAGGAATGAATTG
B1 CYP4C61  121  ..........................G.................................
CYP4C61v1   121  ..........................G.................................
CYP4C61v2   115  ............................................................

BY CYP4C61  181  ATGAAAATGAACAGTGAAAAAAGAGAGTTATACAAATCAATCTACTTGGAATGGTCAGGT
B1 CYP4C61  181  ............................................................
CYP4C61v1   181  ............................................................
CYP4C61v2   175  ............................................................

BY CYP4C61  241  CCATTTGCTGAAATCCATCTTCTAAGGCCAGAATATGTTGAGGTCGCATTGAAAAGTACT
B1 CYP4C61  241  .....................................................G......
CYP4C61v1   241  ...............................................G.....G......
CYP4C61v2   235  ............................................................

BY CYP4C61  301  GTCAACATAACAAAATCAATGGCTTATGACTTCCTTCATGACTGGCTTGGAACTGGTCTA
B1 CYP4C61  301  .............................T..............................
CYP4C61v1   301  .............................T..............................
CYP4C61v2   295  ............................................................

BY CYP4C61  361  CTCACAAGCACAGGCAGAAAATGGCAAGAACGACGTAAAATGATCACTCCTGCTTTTCAT
B1 CYP4C61  361  ..............................A.............T...............
CYP4C61v1   361  .........T....................A.............T...............
CYP4C61v2   355  ..............................A.............T...............

BY CYP4C61  421  TTCGGGATCCTGGAGGATTTTGTCGAGATATTTGGAGAGAAGAGCAGAACTCTTGTAGAA
B1 CYP4C61  421  ........................................................G...
CYP4C61v1   421  ............................................................
CYP4C61v2   415  ............................................................

BY CYP4C61  481  ATATTGAAGAAACAAAAATTCGGAGAGGAATTCGACATGTATCCGATGATAACAAACTGT
B1 CYP4C61  481  .....................................................G......
CYP4C61v1   481  ......................................C.....................
CYP4C61v2   475  ............................................................

BY CYP4C61  541  GCATTGGATATTATTTGCGAATCGGCGATGGGAACCACGGTGAATGCACAGGAGAAAAAG
B1 CYP4C61  541  ..T...................................C.....................
CYP4C61v1   541  ............................................................
CYP4C61v2   535  ............................................................

BY CYP4C61  601  GACTCTGATTATGTGAGAGCGGTCTATGAAGTCAGTGAACTAATACTCTACAGAGCTTTG
B1 CYP4C61  601  ............................................................
CYP4C61v1   601  .......................................T....................
CYP4C61v2   595  ............................................................

BY CYP4C61  661  AGACCATGGCTATATGCAGAGTTTATATGGAAGATGTCAAGTCATGGAAAAGCATTTTAC
B1 CYP4C61  661  ............................................................
CYP4C61v1   661  ...........C................................................
CYP4C61v2   655  ............................................................

BY CYP4C61  721  AGGAATTTGAAAACTCTACATGACTTCACAAATAAAGTAATAGTGGAGCGTAGAGAAGCC
B1 CYP4C61  721  ..................................................A.........
CYP4C61v1   721  ............................................................
CYP4C61v2   715  ............................................................

BY CYP4C61  781  ACTTCTAAAAAATGTTCTCTGAACGAGTCTTACGATGGAGTTGGAAGAAGAAAGAAGAAG
B1 CYP4C61  781  ......................C.......G.............................
CYP4C61v1   781  ..............................G.............~~~~~...~.......
CYP4C61v2   775  ..............................G.............................
```

```
BY CYP4C61  841  GCCTTTCTTGATTTGCTCCTCGAGGCAACTGAAAATGGACATGAGTTATCGCAAGCAGAC
B1 CYP4C61  841  .................A....................T.................TA..
CYP4C61v1   835  .................A..T...................................T...
CYP4C61v2   835  ............................................................

BY CYP4C61  901  ATAAGAGAGGAGGTTGATACATTCATGTTTGAAGGCCACGATACGACGGCAGCAAGCATT
B1 CYP4C61  901  ............................................A........T......
CYP4C61v1   895  ............................................A........T......
CYP4C61v2   895  ............................................A........T......

BY CYP4C61  961  GGCTGGGCGATATTTCTCCTGGGGAACAACCCTGAAGTTCAGGACAGAGTTGTGGAAGAA
B1 CYP4C61  961  ..T.............................A...........................
CYP4C61v1   955  ..T.............................A.......................G...
CYP4C61v2   955  ..T.............................A.................G.....T...

BY CYP4C61  1021 TTGAATGATATCTTTGGTGACTCAGATCGGTTGGCTACCATACATGATCTGAATGACATG
B1 CYP4C61  1021 ............................................................
CYP4C61v1   1015 ............................................................
CYP4C61v2   1015 .................C..T..............C........................

BY CYP4C61  1081 AAATATTTAGAAATGGTCATTAAAGAAACATTAAGGCTCTATCCGAGTGTACCATTCATT
B1 CYP4C61  1081 ..G.....................................................T...
CYP4C61v1   1075 ..G.....................................................T...
CYP4C61v2   1075 ..G.....................................................T...

BY CYP4C61  1141 GGGAGGCTTGTTACTCAAGACATGGTTGTTGGAGAGCATTTAATCCCAGCTGGTGTTTGG
B1 CYP4C61  1141 ...............G............................................
CYP4C61v1   1135 ...............G.G..........................................
CYP4C61v2   1135 ...............G............................................

BY CYP4C61  1201 GTCAACATCGAATTATTTAGCGTGCACAGATGTAGAGATCATTATTCTGATCCAGAAAAG
B1 CYP4C61  1201 ...........G.....C..........................................
CYP4C61v1   1195 .................C...................................T......
CYP4C61v2   1195 ............................................................

BY CYP4C61  1261 TTTAACCCAGACAACTTCCTGCCAGAAAACACGAAAAGTCGACATCCTTTCGCATACGTT
B1 CYP4C61  1261 ..C.........................................................
CYP4C61v1   1255 ..C.........................................................
CYP4C61v2   1255 ............................................................

BY CYP4C61  1321 CCATTTAGTGCTGGTCCCAGAAACTGCATTGGCCAAAAATTTGCTCTACTTGAAGAGAAG
B1 CYP4C61  1321 ...............................................C........A...
CYP4C61v1   1315 .............................A..............................
CYP4C61v2   1315 ...............................................C........A...

BY CYP4C61  1381 ACTATCCTATCTTCTATCTTGAGGAAATTCAGAGTAGAATCGACTGAAAAGCAAGAGGAT
B1 CYP4C61  1381 ..............C..A.................G........................
CYP4C61v1   1375 ............................................................
CYP4C61v2   1375 ..............C..A.................G........................

BY CYP4C61  1441 ATCTGCTTAATGATGGATCTTGTTCTTCGACCAGAGTCTGGCGTCAAAATCAAAATGTAT
B1 CYP4C61  1441 .....TC.T...................................T...............
CYP4C61v1   1435 ............................................................
CYP4C61v2   1435 ............................................T...............

BY CYP4C61  1501 CCTAGAGAACAATAA
B1 CYP4C61  1501 ...............
CYP4C61v1   1495 ...............
CYP4C61v2   1495 ........CA.TA.CAAACAATAA
```

Figure S4. Alignment of *CYP4C61* gene open reading frame sequences of biotype Y and other BPH *CYP4C61* sequences. Single-nucleotide polymorphisms are indicated. Identical nucleotides are represented by dashes. BY CYP4C61: biotype Y

CYP4C61 sequence; B1 CYP4C61: biotype 1 *CYP4C61* sequence; CYP4C61v1: BPH *CYP4C61* sequence from GenBank (FM163384.1); CYP4C61v2: BPH *CYP4C61* sequence from GenBank (KM217037.1).
