# Supplementary material for: Functional Study of Cytochrome P450 Enzymes from the Brown Planthopper (Nilaparvata lugens Stål) to Analyze Its Adaptation to BPH-Resistant Rice
Source: Front Physiol. 2017 Nov 30;8:972. doi: 10.3389/fphys.2017.00972 (PMC5714877; doi:10.3389/fphys.2017.00972)
Supplement: Supplementary file 2 [file Table1.PDF]

Supplementary materials

Table S1. Identification parameters of rice sheath ethanol extract metabolites.

NO.	Rt (min)	Compounds	PubChem CID	NIST matching（%）	Area (%) [a]
1	3.95	2-Pentanone, 4-hydroxy-4-methyl-	31256	83.64	0.06
2	13.33	Benzeneethanamine, N-(1-methylethylidene)-	550985	87.49	0.01
3	13.65	2-Methoxy-4-vinylphenol	332	53.58	0.07
4	14.27	Naphthalene, 1,2,3,4-tetrahydro-1,1,6-trimethyl-	68057	69.33	0.11
5	14.81	1-(3,6,6-Trimethyl-1,6,7,7a-tetrahydrocyclopenta[c]pyran-1-yl)ethanone	605654	80.18	0.08
6	15.70	Cholestan-3-ol, 2-methylene-, (3 á,5 à)-	281906	42.44	0.03
7	16.24	Butylated Hydroxytoluene	31404	81.42	2.49
8	16.73	1,6-Dioxacyclododecane-7,12-dione	13064	70.30	0.16
9	18.80	5,8,11-Heptadecatriynoic acid, methyl ester	582271	64.42	0.05
10	18.88	4-((1E)-3-Hydroxy-1-propenyl)-2-methoxyphenol	9983	82.12	0.04
11	19.01	Tetradecanoic acid	11005	66.27	0.56
12	20.25	3,7,11,15-Tetramethyl-2-hexadecen-1-ol	145386	59.01	7.87
13	20.39	Vitamin E	14985	75.93	0.19
14	20.76	7,9-Di-tert-butyl-1-oxaspiro(4,5)deca-6,9-diene-2,8-dione	545303	87.19	0.36
15	21.33	Hexadecanoic acid, ethyl ester	12366	51.49	0.02
16	22.80	9,12,15-Octadecatrienoic acid, (Z,Z,Z)-	5280934	46.68	4.13
17	22.93	Octadecanoic acid	445639	71.77	0.22
18	23.96	3-Cyclopentylpropionic acid, 2-dimethylaminoethyl ester	91693811	55.40	0.19
19	24.71	9-Octadecenamide, (Z)-	5283387	61.08	0.25
20	25.86	Hexadecanoic acid, 2-hydroxy-1-(hydroxymethyl)ethyl ester	123409	66.12	4.93
21	26.03	Campesterol	173183	65.93	1.56
22	26.86	Stigmasterol	5280794	80.75	5.31
23	28.25	β-Sitosterol	222284	80.53	4.55
24	28.82	Calcitriol	5280453	35.24	0.05

[a] Area percentages (% of total area)

Table S2. Metabolite relative peak area (mean ± SEM) measured by GC–MS from BPH honeydew and the changes in metabolites in honeydew from *GFP*, *NlCPR* and *CYP4C61* dsRNA-treated BPH, respectively, when feeding on YHY15 rice.

Metabolites	Rt (min)	ds*GFP*	ds*NlCPR*	ds*CYP4C61*
**Sugars**				
18 D-Ribofuranose	19.85	0.045 ± 0.027	0.068 ± 0.013	0.091 ± 0.021
22 Fructose	21.89	10.48 ± 3.00	13.56 ± 3.17	8.51 ± 2.23
23 Glucose	22.43	5.47 ± 2.61	8.57 ± 1.46	10.26 ± 4.24
24 D-Galactose	23.08	0.0084 ± 0.0037	0.056 ± 0.019 ↑	0.019 ± 0.0015 ↑
30 α-D-Glucopyranoside	35.06	8.22 ± 4.48	9.53 ± 1.95	12.55 ± 2.76
**Organic acids**				
1 Oxalic acid	5.17	0.074 ±0.016	0.39 ± 0.14	0.19 ± 0.041 ↑
4 Phosphate	8.16	1.30 ± 0.27	1.06 ± 0.20	1.35 ± 0.49
6 Succinic acid	8.90	0.032 ± 0.017	0.062 ± 0.027	0.044 ± 0.013
9 Malic acid	13.22	0.17 ± 0.076	0.14 ± 0.090	0.053 ± 0.026
11 α-Hydroxypyruvic acid	15.06	0.030 ± 0.0067	0.033 ± 0.018	0.010 ± 0.0045 ↓
16 Trans-Aconitic acid	19.10	0.017 ± 0.0041	0.020 ± 0.0064	0.056 ± 0.014 ↑
19 Shikimic acid	20.38	0.20 ± 0.071	0.063 ± 0.014	0.063 ± 0.015
20 1,2,3-Propanetricarboxylic acid	20.59	0.80 ± 0.35	1.11 ± 0.24	1.70 ± 0.43
**Fatty acids**				
25 Hexadecanoic acid	24.53	1.21 ± 0.31	1.97 ± 0.12 ↑	2.37 ± 0.38 ↑

29 Hexadecanoic acid,(2S)-2,3-dihydroxypropyl ester	33.62	0.79± 0.21	1.23 ± 0.10	1.48 ± 0.13↑
28 Octadecanoic acid	28.01	1.11 ± 0.27	1.94 ± 0.10↑	2.14 ± 0.31↑
31 Octadecanoic acid, 2,3-dihydroxypropyl ester	36.39	0.53 ± 0.22	1.14 ± 0.079↑	1.48 ± 0.11↑
**Amino acids**				
2 Valine	6.67	0.31 ± 0.19	0.028 ± 0.0087	0.031 ± 0.02
5 Glycine	8.70	0.061 ± 0.030	0.018 ± 0.015	0.017 ± 0.010
7 Serine	10.12	0.59 ± 0.20	0.057± 0.032↓	0.050 ± 0.034↓
8 Threonine	10.75	0.51 ± 0.31	0.024 ± 0.012	0.040 ± 0.032
10 Proline	13.91	1.75 ± 0.53	0.22 ± 0.095↓	0.33 ± 0.12↓
13 Phenylalanine	16.27	0.43 ± 0.22	0.032 ± 0.016	0.046 ± 0.045
14 Asparagine	17.36	0.48 ± 0.19	0.021 ± 0.0016↓	0.049 ± 0.011↓
15 α-Aminoadipic acid	18.27	0.040 ± 0.018	0.011 ± 0.0026	0.012 ± 0.0038
12 Ornithine	16.02	0.039 ± 0.010	0.0033 ± 0.0012↓	0.0026 ± 0.0012↓
17 Glutamine	19.51	1.47 ± 0.52	0.11 ± 0.017↓	0.23 ± 0.11↓
21 Aspartic acid	20.75	0.048 ± 0.012	0.088 ± 0.0046↑	0.096 ± 0.013↑
27 Tryptophan	27.85	0.10 ± 0.081	0.013 ± 0.0090	0.034 ± 0.030
**Polyols**				
3 Glycerol	8.02	0.27 ± 0.16	0.065 ± 0.019	0.13 ± 0.036
26 Myo-Inositol	25.84	0.31 ± 0.11	0.32 ± 0.068	0.45 ± 0.072

ds*GFP*: *GFP* dsRNA-treated BPH; ds*NlCPR*: *NlCPR* dsRNA-treated BPH; ds*CYP4C61*: *CYP4C61* dsRNA-treated BPH.

↑, significant increase compared with the control (t-test, $P<0.05$); ↓, significant decrease compared with the control (t-test, $P<0.05$).

Table S3. Information about the primers used in this study.

Primer Name	Forward primer (5'-3')	Reverse primer (5'-3')
actin 1	GACAGGATGCAGAAGGAAATCA	GACTCGTCGTACTCCTGCTTTG
GAPDH1	CGTATTGGACGTCTGGTCCT	CCGTGGGTCGAGTCATACTT
CYP3A25	GCAGTGCATCACCTTGAAGA	GTGTGTGGATTGCGTTTGTC
CYP301B1	ATGTGTCTCGGCAGGCGATTC	AACAGTTCTGTCAGTGAATTTGAA
CYP418A1	TTTCAGTTGAAGCGAGGACA	CCCGTCTCTTCCAATAACCA
CYP6CW1	GCTCGGGCTGATAGTGACA	GATCGATCCTGGTGGTAGGA
CYP6CS1	AGGACCACGATTCTGTTTGG	GTTGGTGCTGATTTCCCAGT
CYP4CE1	TAACAATGGACGACCTGCAC	TGCAGTCGCCTAGTCATCAT
CYP303A1	ACACTCTGTGGACCATGCTG	ATTGGCTGAAAAGAGCTCCA
CYP4C62	TGCTGGTATCTGCTGGTCTC	GAGGCAGATTTGAATCACCAA
CYP4C61	GCTGGTGTTTGGGTCAACAT	AATGGAACGTATGCGAAAGG
CYP6AX1	CGCTTCAAGGTGAGGACTTT	CGTTGACCAGGTTGATGAAA
CYP6AY1	TGCTGAGGCAGAAGATTTCA	GACGTCACGCATTTCCAGTA
CYP380C10	TTTTCGTGCCCTCTCACTCT	AGTGTACTTCCGGCTCCATC
CYP404B2	GTGGAACCACAAACGGTCTT	TATGGACCCATAGTGCGTGA
CYP417A2	CGTCAAACCACTGCATGACT	TGAACTGCTCGAGAATGGTG
CYP315A1	TGGCAGACGAATGAGTGAAG	TAGAAGTCGCACCGGAGTTT
CYP18A1	ACGCCAAATCGTCTCAGTTT	CGTTCAGCAAGGAGGAGTTC
CYP404B2	ATGGCGAGATGGAAAAATTG	CACTTGGGAGTGGGGAAGTA
CYP302A1	ATCCTAAACACCCCCTGGTT	CTCGAGCACCATGTGACTGT
CYP314A1	GCCGCCATCATTGACTTTAT	CCTGAGGGGATCACAGACAT
CYP427A1	TCGTATGTGCCCTGGTATGA	TCGGTTTGAGCTCCTCATTT
CYP4G115	GCTGAAGCTGAAGGTGATCC	GGCTGCAGCTTGATTCTGA
CYP380C10	ACTATGATGGTCGGGGGAAG	TATACGCGGTCCTGGATTTC
CYP6FJ2	ACCTGGAAGCGAATTAGTGA	TTAAATTTGTCCGGCTCAGG
CYP417A1	ATAGGCAACGCTCACCTGTT	GGACCGAGCCACACTCTAAC
CYP6ER1	ATTCCGGTCTATGCGCTTC	TGGATTGGCGCTCTCTTACT

Primer Name	**Primer sequence** (5'-3')
dsCYP4C61F	TAATACGACTCACTATAGGGAGATGGCTTGGAACTGGTCTACTCAC
dsCYP4C61R	TAATACGACTCACTATAGGGAGAGCCTTCTTCTTCCCAACTCCATC
dsCYP6AX1F	TAATACGACTCACTATAGGGAGACGAGAAATCAAACAAAAAAGAGT
dsCYP6AX1R	TAATACGACTCACTATAGGGAGACTTGTATCTCTTGGTTGACTGCC
dsCYP6AY1F	TAATACGACTCACTATAGGGAGATCACCTCTGGAAAACTAAAAGCC
dsCYP6AY1R	TAATACGACTCACTATAGGGAGAATTAGCCTCCGTTCCATCATAGT
dsCPRF	TAATACGACTCACTATAGGGAGAAGTCAGACGGGCACGGGAGA
dsCPRR	TAATACGACTCACTATAGGGAGACCACCTCCCCTGTGTAGACT
dsGFPF	TAATACGACTCACTATAGGGAGAGCGAGGGCGAGGGCGATGCC
dsGFPR	TAATACGACTCACTATAGGGAGACTTTGCTCAGGGCGGACTGGG
